# Modulating Peptide Self‐Assembly via Triblock Chiral Patterning

**DOI:** 10.1002/chem.202404603

**Published:** 2025-06-05

**Authors:** Conor L. O'Neill, Jonathan L. Fascetti, Zoe Clapacs, Lauren K. Kaplita, Chih‐Yun Liu, Darren Kim, Mark A. White, Jai S. Rudra

**Affiliations:** ^1^ Department of Biomedical Engineering, McKelvey School of Engineering Washington University in St. Louis St. Louis MO 63130 USA; ^2^ Sealy Center for Structural Biology and Molecular Biophysics and Department of Biochemistry and Molecular Biology University of Texas Medical Branch Galveston TX 77555 USA

**Keywords:** chirality, peptide self‐assembly, supramolecular morphology

## Abstract

The suprastructural integrity of peptide self‐assemblies is driven by an intricate array of cohesive interactions that guide and maintain a hierarchical order. Seemingly minor alterations to atomic arrangement, such as substitution with D‐amino acids, can dramatically affect assembly potential and resultant architecture. When a primary sequence is comprised of consecutive identical motifs, “block heterochiral” peptides can be generated by partitioning chiral inversions according to these underlying elementary units. In this work, we present a combinatorial exploration of all triblock chiral patterns for the model β‐sheet‐forming peptide KFE12 (Ac‐(FKFE)_3_‐NH_2_). Analysis of the four resulting enantiomer pairs reveals that each produces a unique morphology, ranging from minimal 4‐nm‐wide fibrils to micron‐scale semi‐structured aggregates. Our investigation of these variants illustrates a combination of conserved and divergent hierarchical features, reflecting complex interplay between persistent fundamental forces and the unique spatial implications of blockwise intramolecular chiral interfaces.

## Graphical abstract

Chiral block patterning influences peptide self‐assembly by directing molecular orientation, which results in distinct morphologies and packing configurations. This alters the gel's mechanical properties, thereby impacting its applications in tissue engineering and drug delivery. Our research shows that integrating chiral motifs can modify peptide nanostructures, providing flexible strategies for tailoring functional biomaterials.

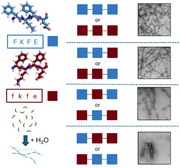

## 1 Introduction

For the purposes of structural exploration, the intrinsic modularity of self‐assembling peptides facilitates and greatly simplifies intentional design and iterative variation.^[^ ^1^, ^2^ ^]^ This tractability enables researchers to refine and amplify particularly influential domains from natural proteins. This is exemplified by a yeast‐derived β‐sheet‐bilayer (“cross‐β”) peptide with alternating polar and nonpolar residues.^[^ ^3^ ^]^ When phenylalanine is selected as the nonpolar component, long‐range aromatic π‐stacking within the hydrophobic core improves fibril cohesion; similarly, solubility and electrostatic association can be enhanced by selecting a charge‐complementary pair of solvent‐exposed residues.^[^ ^4^ ^]^ These alterations produce virtually optimized cross‐β peptides such as those of the KFE family, Ac‐(FKFE)_n_‐NH_2_.^[^ ^5^ ^]^

The collective strength of these integral forces permits substantial primary sequence modification without precluding self‐assembly, affording the production of expansive variant arrays.^[^ ^6^ ^]^ While amino acid composition and patterning is inherently ubiquitous in the field of peptide self‐assembly, de novo design has largely been confined to the use of L‐amino acids.^[^ ^7^ ^]^ In recent years, several research groups have begun utilizing “nonnatural” D‐amino acids in self‐assembling peptide sequences.^[^ ^8^ ^]^ Full substitution predictably yields mirror‐image structures; however, *partial* substitution has proven to be a potent supplement to traditional means of modification.^[^ ^9^, ^10^, ^11^ ^]^ Inclusion of both L‐ and D‐residues produces unique spatial arrangements and side‐chain interactions due to the internal strain imposed on the peptide backbone by atypical H‐bond networks and steric interactions. In tandem with conventional design elements, this powerful strategy redefines the limits of biomolecular customization.

The dramatic influence of partial D‐substitution on secondary structure, morphology, gel mechanics, and bioactivity has been demonstrated for numerous peptide classes.^[^ ^10^, ^12^, ^13^ ^]^ Further, racemic mixtures of all‐L and all‐D self‐assembling isomers stereocomplex into a singular coassembled architecture.^[^ ^14^, ^15^, ^16^ ^]^ At the intersection of these two concepts, “block heterochirality” exploits repetitive sequences (e.g., (FKFE)_n_) to produce mixed‐chiral species that preserve the stereoselective potential of each individual repeat unit. We previously reported on the striking structural departure of the diblock heterochiral KFE8 analogs LD (Ac‐FKFEfkfe‐NH_2_) and DL (Ac‐fkfeFKFE‐NH_2_) from their homochiral counterparts, LL and DD.^[^ ^17^ ^]^ As opposed to β‐sheet bilayers, LD and DL adopt a monolayered structure with width and helical pitch an order of magnitude larger than those of the narrow, tightly wound LL and DD cross‐β fibrils.

**Figure 1:**
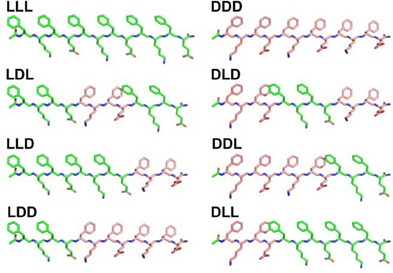
Structures of the eight triblock chiral KFE12 isomers. Carbons are shown in green for L‐residues and in pink for D‐residues.

Motivated by these results, the current work aims to expand the scope of block chirality to KFE12, Ac‐(FKFE)_3_‐NH_2_. Notably, KFE12 has been investigated in characterizing the predominant forces of assembly,^[^ ^18^ ^]^ however, the system has not been studied in the context of block heterochirality. As triblock systems, the eight KFE12 analogs reported in this study (Figure 1) introduce a third variably chiral domain whose nonterminal position isolates its impact from edgeinteractions with laterally associated β‐sheets.

**Figure 2:**
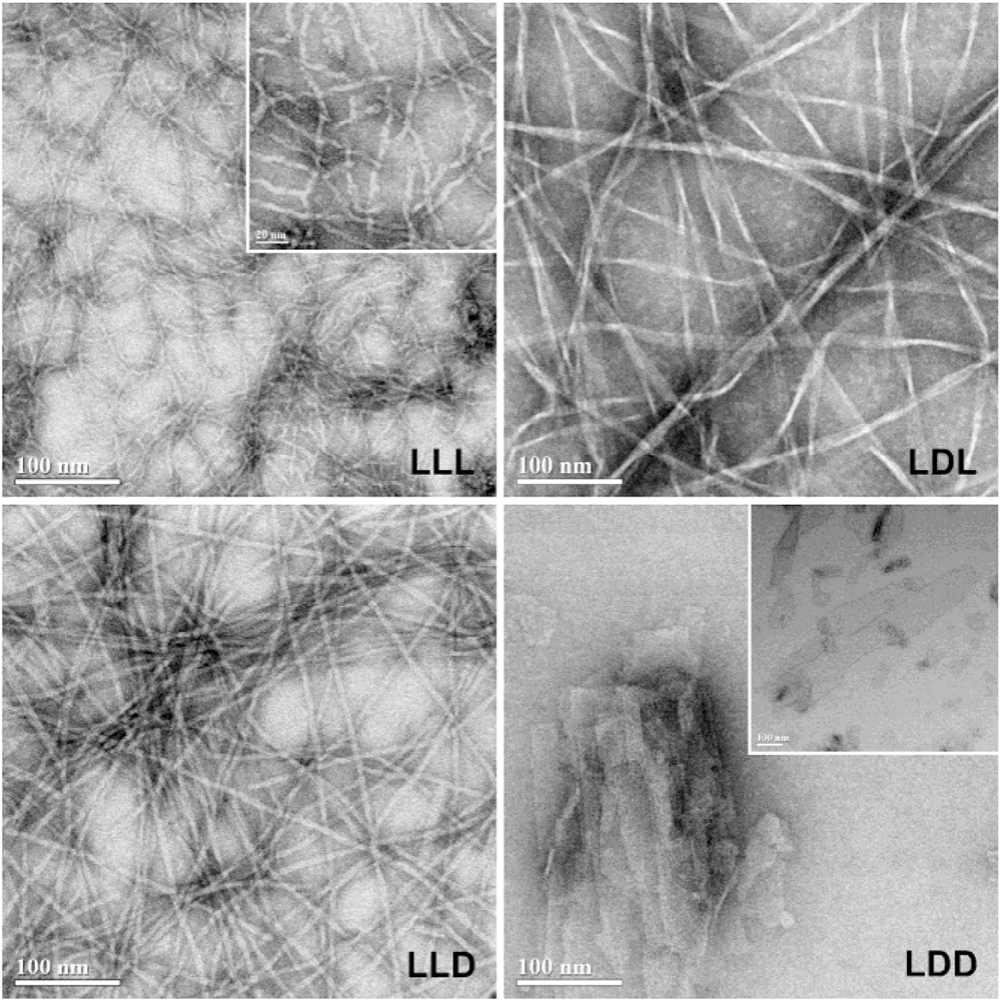
Representative scanning transmission electron microscopy images (800k) for LLL, LDL, LLD, and LDD (enantiomers shown in Figure S1). LLL inset shows a high‐magnification image (1200k, 20‐nm scale bar) with MD‐predicted helicity visible as slight oscillations in width. LDD inset shows an alternate, fragmented morphology (400k, 100‐nm scale bar).

As observed by electron microscopy, the eight triblock chiral analogs adopt four remarkably distinct morphologies (Figure 2), with each pair producing visually indistinguishable suprastructures aside from helical handedness, if present (Figures S1–S3). The homochiral analogs, LLL and DDD, adopt minimal cross‐β fibrillar structures just one peptide in width (4.4 ± 0.4 nm, Figure S2). Though well‐spaced regions were located and imaged, as shown in Figure 2, this morphology often presented as the densely entangled mats pictured in Figure S1. EM‐based visualization of LLL and DDD fibril twists is limited by their comparable diameter (4.4 nm, Figure S2) and plate thickness (3.0 nm, Table S1), with the helicity predicted by energy‐minimization modeling (Figure S9C) visible only in high‐magnification micrographs (Figure 2, inset). By contrast, LDL and DLD helical folds were made readily apparent by their increased diameter (12.5 ± 3.0 nm, Figure S2) and pitch (259 ± 45 nm, Figure S3). Reminiscent of heterochiral KFE8, the extent of lateral β‐sheet aggregation is variable, with LDL and DLD fibrils ranging from two to five peptides in width (Figure S2). When compared to LDL and DLD, fibrils formed by LLD and DDL are entirely flat and approximately half as wide (6.3 ± 0.7 nm). In a significant departure from the otherwise fibrillar tendencies of their isomers, LDD, and DLL instead form irregular, 2D cross‐β sheets with diameters ranging from 5 to 200 nm. Beyond individual sheets, multi‐layered 3D aggregates with diameters up to 4 µm were observed in multiple independently prepared samples. This indicates that the smaller structures commonly observed alongside larger sheets (inset) may be fragments of assemblies fractured during solubilization and grid adsorption.

**Figure 3:**
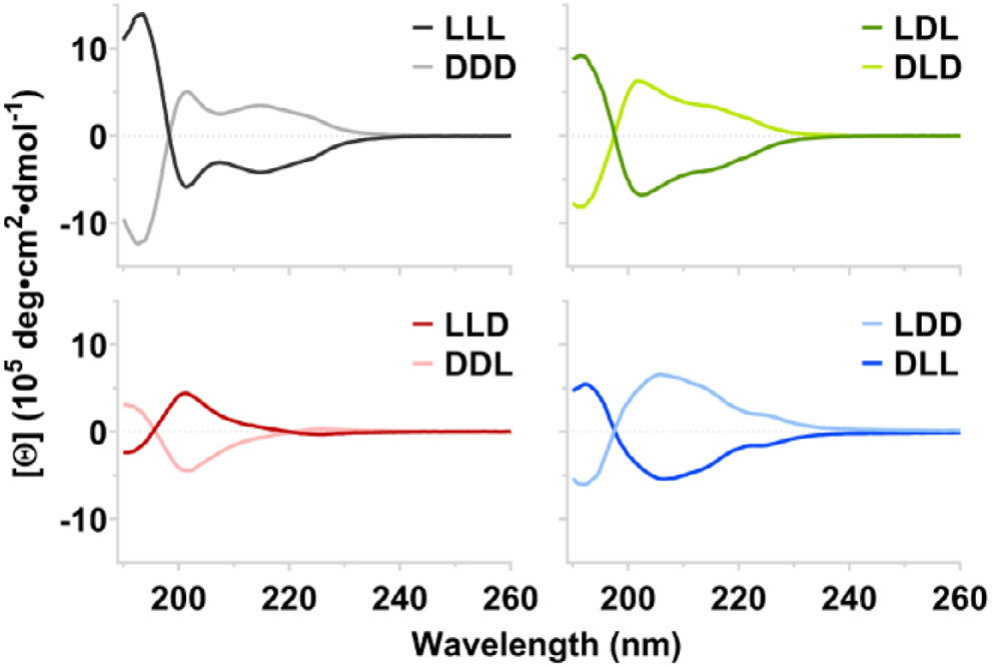
Circular dichroism spectra for the KFE12 analogs (0.10 mM) in water at room temperature. CD signs follow the chirality of their C‐terminal blocks, and β‐sheet character is evident in all samples.

Despite these substantial differences, circular dichroism (CD) (Figure 3) and Fourier‐transform infrared spectroscopy (FTIR) (Figure S4) reveal that all analogs share antiparallel cross‐β secondary structure. In agreement with EM data, enantiomer pairs produce mirror‐image CD spectra, confirming that they differ only in handedness. As we have previously reported, L‐ and D‐homochiral β‐sheets produce negative and positive Cotton effects, respectively, between 240 and 200 nm.^[^ ^17^ ^]^ The inherent imbalance of three‐component binary systems grants all KFE12 isomers either majority‐L or majority‐D chiral composition. As a fundamental, relatively independent structural feature, interstrand H‐bond networks and their associated n → π* transitions (∼222 nm)^[^ ^19^ ^]^ are almost entirely defined by backbone chirality, with sign and magnitude at this wavelength dictated by the proportion of L‐ and D‐ amino acids within each analog. Interestingly, this rule only appears to hold true for the n → π* region. For instance, LLD is majority‐L and, as expected, has a negative sign at ∼222 nm. At lower wavelengths, however, LLD is strongly positive with a prominent phenyl π‐stacking peak at ∼203 nm (π → π*).^[^ ^19^ ^]^ Along with its enantiomer DDL, LLD is the only block chiral KFE analog whose CD spectrum crosses zero in the 260–200 nm range. Irrespective of this unique feature, the principal CD sign of all KFE12 isomers is dictated solely by the chirality of their C‐terminal blocks. The dependence of both these trends on chiral composition and patterning illustrates the complexity of heterochiral interactions, as they hold true for both di‐ and triblock KFE systems.

LLL and DDD appear as classical cross‐β CD signatures with a particularly pronounced trough (∼208 nm) separating their two major peaks (Figure 3). LDL and DLD retain cross‐β character, though their spectra are far more reminiscent of KFE8, with LDL (or DLD) appearing as a near‐perfect superposition of LL and DL (or DD and LD).^[^ ^17^ ^]^ This is particularly intriguing given that the width and pitch of LDL and DLD are intermediate to those of homochiral and heterochiral KFE8. The spectrum of LLD (or DDL) can analogously be described as a superposition of LL and LD (or DD and DL). Given that LLD contains overlapping LL and LD domains (and that DDL contains overlapping DD and DL domains), the opposite handedness of each pair of domains may serve to reduce or effectively eliminate any natural twist from LLD and DDL. The broad, largely featureless β‐sheet signatures produced by LDD and DLL may reflect a multitude of less restrictive packing modes that enable their apparent multidimensional propagation (Figure 2).

**Figure 4:**
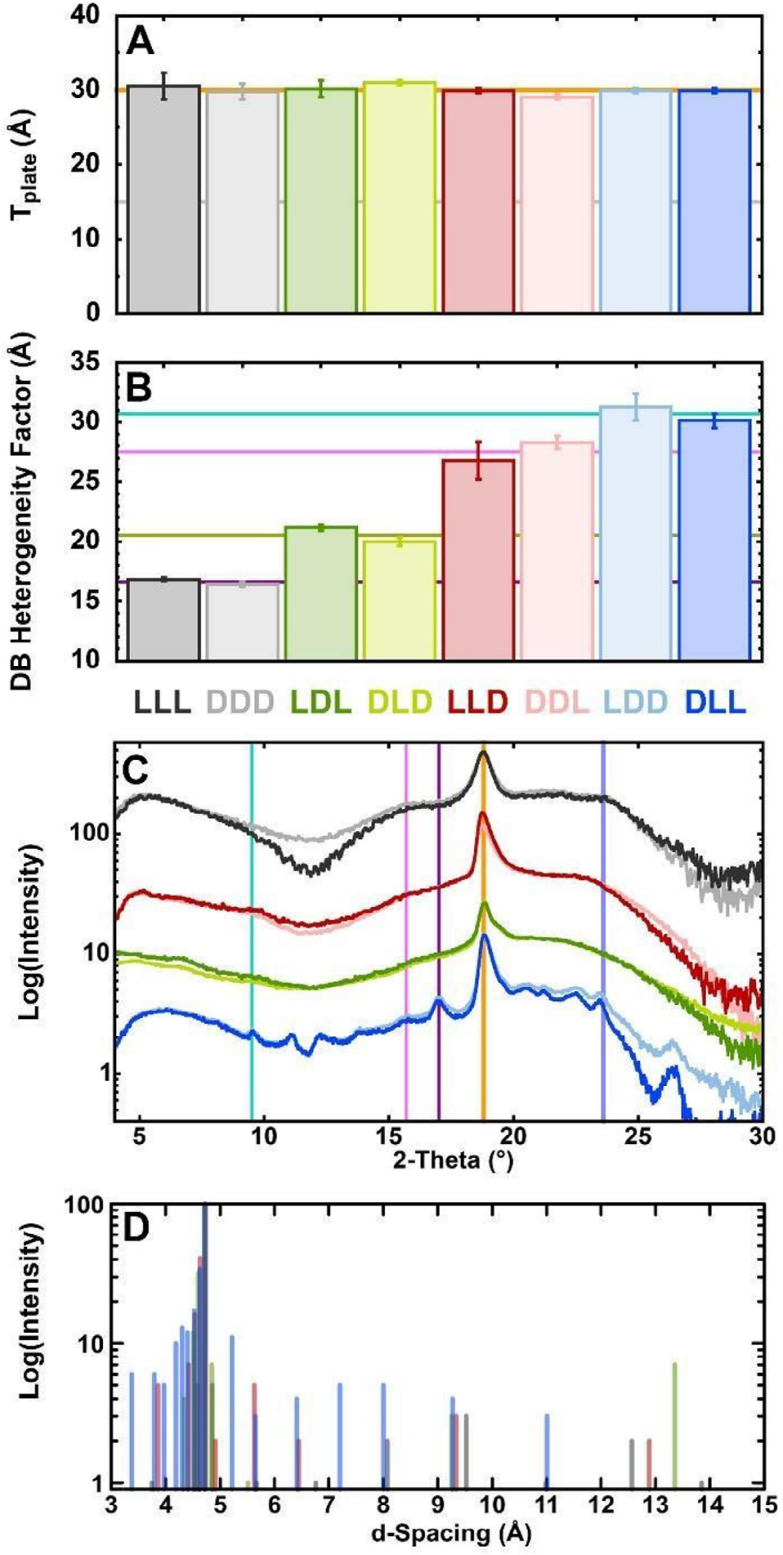
SAXS parameter values, WAXS curves and d‐spacings. A) SAXS plate thickness for each peptide (Figure S6, Table S1). Orange line marks average thickness (30 Å) for all KFE12 peptides. Gray line marks single‐sheet thickness. B) DB heterogeneity values for each analog. Colored lines mark averages for each pair of enantiomers. C) WAXS diffraction curves. Common features are highlighted by colored vertical lines: 2×β‐sheet 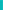 at d = 9.31 Å, A*β*(42) 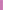 at d = 5.68 Å, a peak unique to LDD and DLL 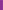 at d = 5.28 Å, β‐sheet 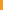 at d = 4.72 Å, and Cα‐Cα 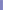 at d = 3.77 Å. Curves offset for clarity. D) WAXS d‐spacings. Error bars in panels A and B were calculated using the asymptotic standard error of the fit to the Guinier equation in Gnuplot.

Small‐angle X‐ray scattering (SAXS) data for each KFE12 isomer and its enantiomer are similar, with only minor discernable differences emerging at low‐q (Figures 4 and S5). Due to their expansive multimodal assemblies and subsequently reduced solubility, LDD and DLL readily precipitate out of the high‐concentration solutions required for X‐ray diffraction studies. Holtzer plot analysis reveals interfilament scattering contributions for all heterochiral isomers, as supported by deviations from linearity at low‐s values in their Guinier Plate plots (Figure S6), though no such scattering contributions are observed for the homochiral isomers LLL and DDD. These were low‐s scattering deviations from the Guinier and Holtzer models, but were fit by the Debye‐Bueche (DB) model (Figure S7). While these factors affected Guinier radius of gyration (*R*_g_) and cross‐sectional radius (*R*_cs_) calculations (Table S1), they did not affect the higher‐resolution regions (*q* > 0.8/*R*_t_) that are relevant to the Guinier plate analysis, which remained linear.

Despite their substantial morphological and spectroscopic differences, SAXS found that the block chiral isomers of KFE12 have similar underlying structural and assembly profiles. Fibril thicknesses, as measured by Guinier R_plate_ analysis, were found to be virtually identical for all analogs, with *T*_plate_ values ranging from 29.2 to 31.0 Å (Figure S6). Unlike heterochiral KFE8 (LD and DL), which were previously observed to form a monolayer, heterochiral KFE12 analogs retain the two‐molecule thickness of their homochiral counterparts (Figure 4A).

Though SAXS showed that the samples lack the regular intermolecular associations indicative of a greater assembly, high‐concentration solutions produce porous self‐supporting hydrogels consisting of entangled fibrils and interacting regions. The DB model produces a characteristic length (ξ) for each component that describes the spatial arrangement of fibrils and interacting regions (Figures 4B, S7).^[^ ^20^ ^]^ While the similarity between ξ_LDL,DLD_ (33, 30 Å) and ξ_LLD,DDL_ (36, 28 Å) is unsurprising given their comparable morphologies, LDD and DLL produce nearly identical ξ‐values (39, 32 Å) despite their atypical, nonfibrillar architecture. However, the homochiral KFE12 analogs LLL and DDD have far shorter characteristic lengths (16, 16 Å) that correspond to smaller contiguous regions with their gels.

Rather than the network‐scale differences in density depicted by SAXS, wide‐angle X‐ray scattering (WAXS) probes regular atomic arrangements (d‐spacings) within the individual suprastructures that compose these networks resulting in sharp diffraction peaks. KFE12 analogs demonstrate amorphous scattering from the random mixture of filaments in the gel that produces a smooth intense background, which may obscure some of the weaker diffraction peaks. All WAXS curves have broad minima at 2θ‐values of 12° and 29° (Figure 4C), along with a broad maximum in the Cα‐Cα region (23.6°, d‐spacing = 3.8 Å) (Figure 4C, D). Each KFE12 curve is dominated by a major antiparallel β‐sheet diffraction peak at 2θ = 18.8° (4.72 Å) that is asymmetrically widened by several β‐mismatch peaks at slightly higher angles. The closely related 2×β‐sheet peak spacing at 2θ = 9.5° (9.31 Å) is observed for the heterochiral KFE12 isomers, as expected, but is curiously absent in LLL and DDD. Another common feature in cross‐β fibrils, the Aβ_42_‐repeat peak (15.6°, 5.6 Å), is present in all isomers except those with two internal chiral interfaces (LDL and DLD), though these do have a nearby minor d‐spacing at 5.5 Å. Apart from these peaks, most KFE12 WAXS curves are otherwise effectively featureless. In contrast to other isomers, LDD and DLL display a variety of minor peaks with significant intensity (Figure 4D), indicating that their visually amorphous morphology is in fact the product of various coexisting intermolecular selectivities.

To further elucidate differences between the packing modes of KFE8 and KFE12, LLL KFE12 modeling was performed based on X‐ray scattering data and a published model for KFE8 (Hwang, 2003).^[^ ^21^ ^]^ The antiparallel β‐sheets could form in two possible configurations with an equal number of backbone H‐bonds: one in which the acetylated N‐termini are more solvent‐exposed (ACE), and one in which the amidated C‐termini are more solvent‐exposed (CT2). Molecular dynamics simulations of these two initial models found a rapid stabilization of their helical twists at slightly different characteristic values. After 10 ns of fully solvated MD, the ACE and CT2 models settle at ‐9.8° and ‐5.6°, respectively (Figure S8A). Though the nonbonded interaction energies of both models are relatively similar, it was found to be 77 kcal/mol/strand lower for the acetylated N‐termini model (Figure S8B).

The resulting filament model matches the SAXS data in multiple analyses, including its 30‐Å thickness and 16‐Å cross‐sectional radius (Table S1), with χ^2^ values of 3.3 for ACE and 3.7 for CT2. While ensemble modeling with filaments up to 250 nm in length improves χ^2^ to 2.1 (Figure S9), including filaments up to 400 nm long further improves the fit, reducing χ^2^ to 1.7. The ensembles included filaments covering a wide range of lengths, from short protofilaments to filaments up to 400 nm long (Figure S10).

As fibrillar assemblies entangle into hydrogels at high concentration, 10‐mM hydrogels of the triblock chiral KFE12 isomers were analyzed using an HR‐20 rheometer fitted with a 20‐mm smooth parallel‐plate geometry. Three independently prepared replicates of each sample were evaluated with constant parameters set to 0.1 Hz (strain sweeps), 1% oscillatory strain (frequency sweeps), or both (time sweeps). As in previous experiments, LDD and DLL precipitated rather than forming self‐supporting gels and were excluded from subsequent analysis. Time sweeps demonstrated shear recovery for all gelated solutions, reaching stable dynamic moduli within 10 minutes of extrusion through a 22‐gauge needle.

**Figure 5:**
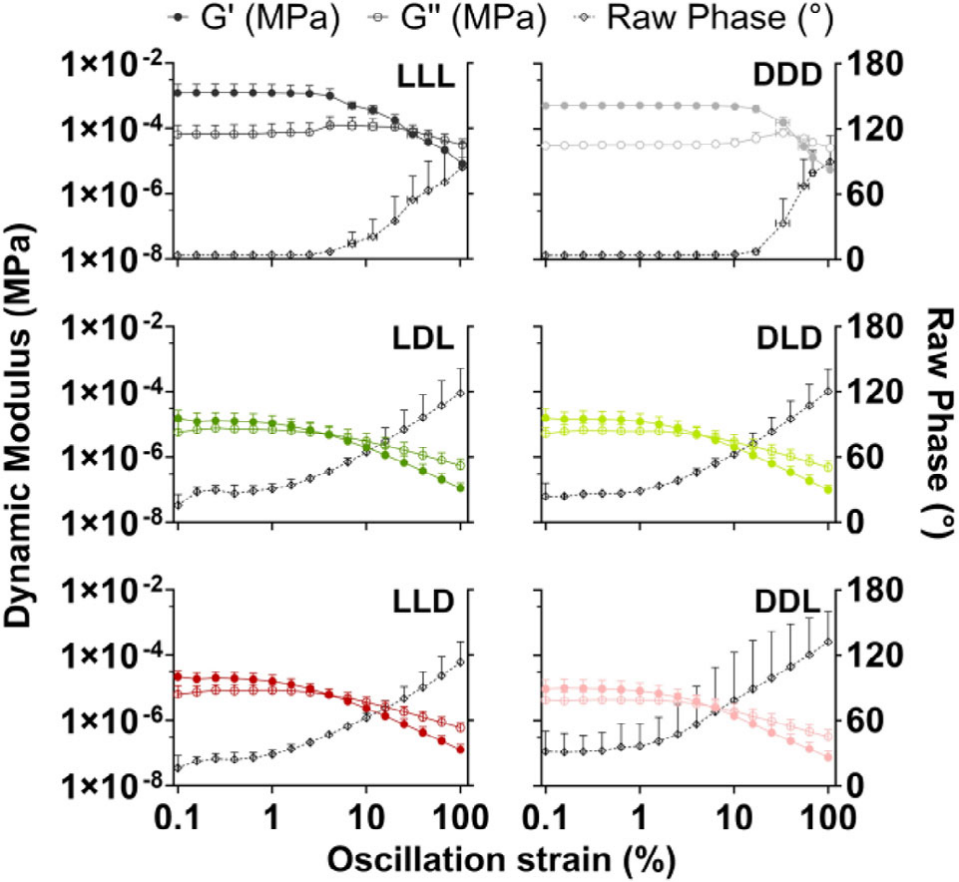
Amplitude sweeps for the hydrogels, with dynamic modulus (MPa) on the left y‐axis and raw phase (°) on the right y‐axis. The raw phase is well below 180° at all points shown, indicating the data is valid and not dominated by momentum of the measurement instrument. The homochiral gels (LLL and DDD) have much higher dynamic moduli (G′ ∼10^−3^ MPa) than the heterochiral gels (G′ ∼10^−5^ MPa). Crossover points are also at greater strains for the homochiral gels. All gels have G′>G″ for low oscillatory strain, indicating that they are viscoelastic solids up to a certain strain, after which they become viscoelastic liquids (G″>G′). Error bars represent the mean ± SEM of n = 3 gels per sample.

Frequency sweeps from 100 to 0.1 Hz had low raw phase (well below 180 degrees), verifying measurement validity below ∼4 Hz for LLL and DDD and below ∼0.6 Hz for LDL, DLD, LLD, and DDL (Figure S11). All formulations remain viscoelastically solid (G′ > G″) for all low‐raw‐phase frequencies. The hydrogels also exhibit shear‐thinning behavior in this range, demonstrating suitability as injectable biomaterials (Figure S12).^[^ ^22^ ^]^ During amplitude sweeps, raw phase remained low for oscillatory strain levels between 0.1% and 100% (Figure 5). Each chiral pair displayed highly similar dynamic modulus profiles across the oscillatory strain levels tested; while minor differences were observed, they were not deemed significant. Crossover points, where gels transition from primarily elastic to viscous behavior, were measured as approximately 31% and 55% for LLL and DDD, 6% for LDL and DLD, and 6% and 10% for LLD and DDL. The linear region, or the strain regime in which phase angle remains effectively constant, was determined to be below 2.5% and 10.5% for LLL and DDD, below 1.0% and 6.3% for LDL and DLD, and below 0.6% and 1.0% for LLD and DDL (Figure S13). It is hypothesized that rheological profiles are not the same between chiral pairs because of different morphological building units that make up the hydrogels. Specifically, the morphologies shown in Figure 2, self‐assembled peptide at a relatively low concentration, give rise to varying rheological properties.

The aim of this report is to highlight the potential of block heterochirality in self‐assembling peptide systems. The power of this method resides in that there is no chemical change to the KFE12 analogs, simply a spatial reorientation of certain amino acids. Expanding from previous efforts, this work showcases how the partial substitution of D‐amino acids in the 12‐mer peptide KFE12 yields unique resultant morphologies. As quantified through EM‐based visualizations, enantiomer pairs exhibit highly similar widths and pitches, in the case of LDL and DLD (Figure S2 and S3). Following, CD analysis showed that all KFE12 analogs have dominant β‐sheet signatures, while enantiomer pairs are mirror images of each other, as expected. X‐ray scattering techniques confirmed β‐sheet dominant structure in the high‐concentration samples. Computational modeling was performed on LLL inspired by previous KFE8 studies, which suggested energy‐minimizing helical twisting angles. Finally, rheological experiments on KFE12 analogs showed that all of the peptides exhibit shear‐thinning behavior and each enantiomer pair has matching dynamic moduli across a range of oscillation strains.

Early studies on the effects of chirality on peptide assembly primarily focused on single‐residue or full‐molecule D‐substitutions. The recent emergence of patterned chiral inversion as a complementary design tool gives rise to unique intramolecular selectivity that fundamentally alters the behavior of well‐established model systems. By applying this concept to a simple, repetitive sequence such as KFE12, we illustrate the widely diverse influence of chiral blocking on material properties ranging from packing mode and supramolecular morphology to hydrogel mechanics. Through a combination of microscopic, spectroscopic, and computational techniques, we describe the complex interplay between enantiomeric building blocks in self‐assembled heterochiral species and the dramatic differences induced by simple rearrangement of these units. This work has further developed our understanding of block chiral patterning and confirmed that this strategy can be extended beyond two‐component systems, supporting the potential for generalization to other self‐assembling peptide classes. In concert with existing design elements, ordered chiral patterning provides an exponentially broader set of building blocks from which to construct synthetic molecular assemblies, representing a simple yet incredibly powerful tool for the creation and tailoring of custom biomaterials.

## Supporting Information

Experimental procedures; additional STEM images; width and pitch histograms; second‐derivative FTIR spectra; SAXS analysis plots; WAXS curves and d‐spacings; energy‐minimization plots and visual representations; ensemble fitting data; additional rheological analyses; peptide identities and purities; HPLC chromatograms; MALDI‐TOF‐MS spectra (PDF).

## Conflict of Interests

The authors declare no conflicts of interest.

## Supporting information



Supporting information

## Data Availability

Additional data is available upon request.
