# Supplementary material for: Modulating Peptide Self‐Assembly via Triblock Chiral Patterning
Source: Chemistry. 2025 Jun 5;31(37):e202404603. doi: 10.1002/chem.202404603 (PMC12223464; doi:10.1002/chem.202404603)
Supplement: Supplementary file 1 — Supporting information [file CHEM-31-e202404603-s001.docx]

**Supporting Information**

**Modulating Peptide Self-Assembly via Triblock Chiral Patterning**

Conor L. O’Neill^†^, Jonathan L. Fascetti^†^, Zoe Clapacs^†^, Lauren K. Kaplita^†^, Chih-Yun Liu^†^, Darren Kim^†^, Mark A. White^‡^, and Jai S. Rudra*^,†^

^†^Department of Biomedical Engineering, McKelvey School of Engineering, Washington University in St. Louis, St. Louis, MO 63130, USA

^‡^Sealy Center for Structural Biology and Molecular Biophysics and Department of Biochemistry and Molecular Biology, University of Texas Medical Branch, Galveston, TX 77555, USA

*Email: srudra22@wustl.edu; Fax: (314) 935-7448; Tel: (314) 935-8704

**Experimental Details** S2-S4

**Figure S1**. Representative STEM images for DDD, DLD, DDL, and DLL S5

**Figure S2.** Fibril width histogram for LLL, DDD, LDL, DLD, LLD, and DDL S6

**Figure S3**. Fibril pitch histogram for LDL and DLD S6

**Figure S4.** Second-derivative FTIR spectra and Gaussian deconvolutions S7

**Figure S5**. Small-angle X-ray scattering (SAXS) Log-Log plots S7

**Figure S6**. Guinier fitting of SAXS data S8

**Table S1**. SAXS characteristics of the peptide filaments and gels S8

**Figure S7**. Debye-Bueche fitting of SAXS data S9

**Table S2**. WAXS data collection and results S9

**Figure S8**. ACE and CT2 molecular dynamics models of LLL S10

**Figure S9**. Energy-minimization modeling of LLL KFE12 S10

**Figure S10**. Ensemble fitting of LLL models to X-ray scattering data S11

**Figure S11**. Oscillatory rheology, frequency sweeps S12

**Figure S12**. Frequency sweep complex viscosity S13

**Figure S13**. Oscillatory rheology, amplitude sweep phase angle S14

**Table S3**. Peptide mass and purity S15

**Figure S14.** LLL HPLC chromatogram S16

**Figure S15**. LLL MALDI-TOF-MS spectrum S16

**Figure S16.** DDD HPLC chromatogram S17

**Figure S17**. DDD MALDI-TOF-MS spectrum S17

**Figure S18.** LDL HPLC chromatogram S18

**Figure S19**. LDL MALDI-TOF-MS spectrum S18

**Figure S20.** DLD HPLC chromatogram S19

**Figure S21**. DLD MALDI-TOF-MS spectrum S19

**Figure S22.** LLD HPLC chromatogram S20

**Figure S23**. LLD MALDI-TOF-MS spectrum S20

**Figure S24.** DDL HPLC chromatogram S21

**Figure S25**. DDL MALDI-TOF-MS spectrum S21

**Figure S26.** LDD HPLC chromatogram S22

**Figure S27**. LDD MALDI-TOF-MS spectrum S22

**Figure S28.** DLL HPLC chromatogram S23

**Figure S29**. DLL MALDI-TOF-MS spectrum S23

**References** S24-S25

**Experimental Details**

**Peptide Synthesis**

All peptides were purchased from P3 BioSystems (Louisville, KY) for molecular and spectroscopy studies. Peptides for rheological analysis were synthesized using Fmoc chemistry on a CEM Liberty Blue™ automated synthesizer. After swelling Novabiochem (Burlington, MA) rink amide MBHA (4-methylbenzhydrylamine) resin for one hour, double coupling of residues was performed using Oxyma (ethyl cyano(hydroxyimino)acetate) and DIC (*N*,*N*'-diisopropylcarbodiimide). Peptide cleavage was achieved in a cocktail (95:2.5:2.5) of TFA (trifluoroacetic acid), TIS (triisopropylsilane), and water. Crude products were pelleted via centrifugation, washed 5x in cold diethyl ether, freeze-dried, and stored as powders at -20°C.

**Peptide Purification and Identification**

Crude peptide was dissolved in 1:1 ACN(acetonitrile):TFA and injected onto an Agilent (Santa Clara, CA) semi-preparative UltiMate 3000 high-performance liquid chromatography (HPLC) system employing a reverse-phase Zorbax column (SB-C18, 21.2 x 150 mm, 5 µm). A gradient of ACN/water (0.1% TFA) was flowed at 18 mL/min, with UV absorbance monitored at 214 and 254 nm. After fraction collection and freeze-drying, purity confirmation was obtained on an analytical UltiMate 3000 system employing a reverse-phase Poroshell 120 column (SB-C18, 4.6 x 150 mm, 2.7 μm), followed by matrix-assisted laser desorption/ionization-time-of-flight mass spectrometry (MALDI-TOF-MS) using a α-cyano-4-hydroxycinnamic acid matrix from Bruker (Billerica, MA) to confirm final purified product identity.

**Peptide Self-Assembly**

Purified peptide products were massed on a Sartorius (Göttingen, Germany) Quintex 65-1S semi-micro analytical balance. Stocks were solubilized in a solution of 1:1 ACN/water, separated into aliquots (0.10 mM), lyophilized for 48 hours, and stored as powders at -20°C. Non-gelated experimental samples (STEM, CD, FTIR) were solubilized in water (0.10 mM) and gently pulsed on a Fisherbrand (Hampton, NH) Fixed-Speed Vortex Mixer.

**Scanning Transmission Electron Microscopy (STEM)**

KFE12 peptide solutions (0.10 mM in water) were negative-stained with 7.5% uranyl formate (UF). To prepare staining solutions, UF (37.5 mg) was added to boiled and cooled MilliQ water (5 mL), vortexed for five minutes, pH-balanced with NaOH (20 µL, 2 M), vortexed for an additional five minutes, filtered through a 4-mm PTFE syringe filter (0.2 µm), and aliquoted into individual 0.5 mL conical tubes. UF aliquots were flash-frozen and stored at -80°C. Before use, UF aliquots were fully thawed and placed in an Eppendorf (Hamburg, Germany) 5417C Centrifuge at 25,000 RCF for five minutes, followed by re-filtering the supernatant and storing under foil at room temperature. Ted Pella (Redding, CA) 200-mesh, pure-carbon-coated grids were glow discharged, inverted onto a 10-µL sample droplet for 60 seconds, briefly washed in three consecutive 10-µL MilliQ water wash droplets, then stained for 60 seconds in a 10-µL 7.5% UF droplet. Grids were desiccated for 60 seconds and allowed to air-dry for 15 minutes before storage in a foil-covered grid box. Micrographs were acquired on a JEOL (Akishima, Japan) JEM-2100F Field-Emission STEM using Gatan 805 bright-field/dark-field (BF/DF) and Gatan high-angle annular dark-field (HAADF) detectors with an accelerating voltage of 200 keV, spot size of 0.5 nm, and camera length of 10 cm.

**Width and Pitch Measurements**

A Fiji image processing package was utilized for STEM image analysis and assembly dimension measurements. Length scales were assigned using JEOL software-generated scale bars. Width measurements (n≥150) were obtained from high-magnification micrographs, on which line segments perpendicular to peptide suprastructure edges were superimposed and measured. Helical pitch, when applicable, was quantified in an analogous manner, with segments drawn between the alternating 180° fibril twists at the point of minimal width. Pitch measurements were supplemented by TEM micrographs acquired on a JEOL (Akishima, Japan) JEM-1400 transmission electron microscope with an accelerating voltage of 120 kV.

**Circular Dichroism**

Circular dichroism (CD) spectra of 0.10 mM KFE12 isomer solutions were acquired using a Jasco (Hachioji-shi, Japan) J-815 CD spectrometer using a Hellma (Plainview, NY) quartz cuvette (110-1-40) with a path length of 1 mm. Three accumulations for each sample were obtained between 300 and 190 nm at 50 nm/min with a 0.1 nm data pitch, 1.00 nm bandwidth, and 100 mdeg sensitivity. High-tension voltage (HT[V]) and absorbance were also collected to ensure data quality.

**Fourier-Transform Infrared Spectroscopy**

Attenuated total reflectance-Fourier transform infrared (ATR-FTIR) spectra were measured with a Bruker (Billerica, MA) Alpha II FTIR outfitted with a zinc selenide crystal. For each analog, lyophilized peptide was gelated and subsequently diluted to 15 mM in ultrapure DI water. Standardized background and solvent spectra were acquired prior to collecting each absorbance spectrum. Spectra were generated from 24 accumulations each using a 4 cm^-1^ step size. Data processing was performed in GRAMS/AI (Thermo Scientific, Waltham, MA). After background subtraction, a Savitzky-Golay filter (third order, nine-point window) was used to calculate second-derivative spectra. The resulting trace was inverted and truncated to 1710–1610 cm^-1^. Baseline correction was performed assuming flat baseline segments between minima in this range. Component peak identification and fitting in the 1710–1640 cm^-1^ domain was performed using Gaussian curves with a full width at half maximum (FWHM) height of eight cm^-1^ and medium sensitivity level, as informed by Akaike Information Criterion (AIC). The peaks of the fitted curves were then compared to secondary structure ranges as described in the literature.^1–3^

**Small- & Wide-Angle X-ray Scattering**

SAXS data of the KFE12 solutions were collected using a Rigaku (Woodlands, TX) BioSAXS-1000 camera on an FRE^++^ X-ray generator with an ASC-96 Automated Sample Changer held at 10°C. A matching buffer was collected for each sample. The detector was calibrated using a silver behenate powder sample, following the manufacturer’s recommended procedure. The KFE12 SAXS samples were prepared by dissolving the lyophilized powders in ddH_2_O. LLL was prepared at 1.6 mM; DDD and LLD at 3.3 mM; and LDL, DLD, DDL, LDD, and DLL at 5.0 mM. Centrifugation was performed to ensure proper mixing of the sample and solute. Samples were vortexed to re-suspend larger particles. Although the samples formed gels, these remained fluid under hydrostatic pressure, permitting pipetting. The samples did not display concentration dependence, and analysis used the highest-concentration SAXS data collected (Table S1). Processing was performed in SAXSLab (Rigaku) and SAXNS-ES (https://xray.utmb.edu/SAXNS). Analyses were performed in Primus/GNOM^4^, BIFT^5,6^, and gnuplot (http://www.gnuplot.info). Comparisons of the Molecular Dynamics-based filament models with the SAXS data were performed using Crysol^7^ and EOM^8^.

WAXS data were collected at room temperature using 10 mM KFE12 solutions and matching buffers. Solutions were pipetted into a 2-mm MiTeGen (Ithaca, NY) MicroRT polyester capillary on a Rigaku R-AXISIV^++^ and processed using Fit2D, SAXNS-ES, and Primus. WAXS was performed with a nominal detector distance of 150 mm and 250 mm. The sample-to-detector distances were calibrated using a MicroRT capillary filled with powdered sucrose. The sucrose cell dimensions were determined using a Bruker D8/TXS diffractometer with a Cu X-ray source. Diffraction data were collected to d=0.84 Å from a single crystal of sucrose (0.2 x 0.2 x 0.2 mm) with the same X-ray source, and the structure was solved using ShelXT. The sucrose cell dimensions were determined to be a=7.943(3) Å, b=8.718(4) Å, c=10.861(4) Å, β=103.05(1)°. These cell dimensions are within the range observed in previous publications.^9–12^ WAXS curve fitting was performed in Primus and gnuplot, and WAXS local background subtraction used second-degree polynomials. Peak fitting used a common Gaussian peak width with geometric correction. Theoretical WAXS curves were generated from models using FoXS^13^ and WAXSiS^14^.

**Molecular Dynamics Modeling**

KFE12 filament models were created using COOT and PyMOL, loosely based on the published KFE8 models (Hwang, 2003).^15^ Standard antiparallel β-sheet stereochemistry for the KFE12 peptide was imposed in COOT, along with standard interstrand hydrogen bonding for both sheets. A flat, 12-strand-long, 2-start sheet was created by translation of the COOT model. The PSF and PDB files for NAMD were created using the VMD psfgen, solvate, and autoionize plugins. Each model was then minimized and annealed in NAMD^16^ before performing a 10-ns molecular dynamics simulation with implicit solvent and 2-fs time steps. These initial models were extended to 44-chain filaments and then fully solvated in 150 mM NaCl. These extended models were energy minimized and annealed prior to 10-ns molecular dynamics runs performed with the CHARMM36 forcefield and the TIP3P water model. Analysis of the NAMD simulations was performed using the VMD^17^ interface and TCL scripts, including the NAMDEnergy plugin. For SAXS/WAXS analysis, the filament models were extended in PyMOL to create multi-turn filaments of increasing length up to 400 nm. This procedure was followed for the two possible inter-strand hydrogen bonding patterns, with either acetylated N-termini or amidated C-termini more solvent-exposed.

**Rheology**

For rheological testing, hydrogels were formed by dissolving each KFE12 analog at 17.2 mg/mL in DI water. The sample was then extruded through a needle onto an HR-20 rheometer fitted with a 20-mm-diameter flat geometry, followed by lowering the geometry to a gap height of 500 µm. Each gel was centered and sheared to failure by rotation at 1 radian/second for a minimum of 10 seconds. The rotation was then stopped and the test sequence was initiated, consisting of a 5-minute time sweep with a frequency of 0.1 Hz and an oscillatory strain of 1%. The gel was then allowed to rest for 30 seconds before a frequency sweep ranging from 100 to 0.1 Hz with oscillatory strain held constant at 1%. After conclusion of the frequency sweep, a strain sweep was performed with oscillatory strain ranging from 0.1% to 100% and frequency held constant at 0.1 Hz. This process was repeated three times for each gel formulation, with each repeat corresponding to an independently prepared gel. During rheological testing, the storage modulus (G′) and loss modulus (G″) were recorded, as well as the phase angle (δ) and raw phase (°) as an indication of data quality. Complex viscosity was computed from storage and loss moduli and frequency.


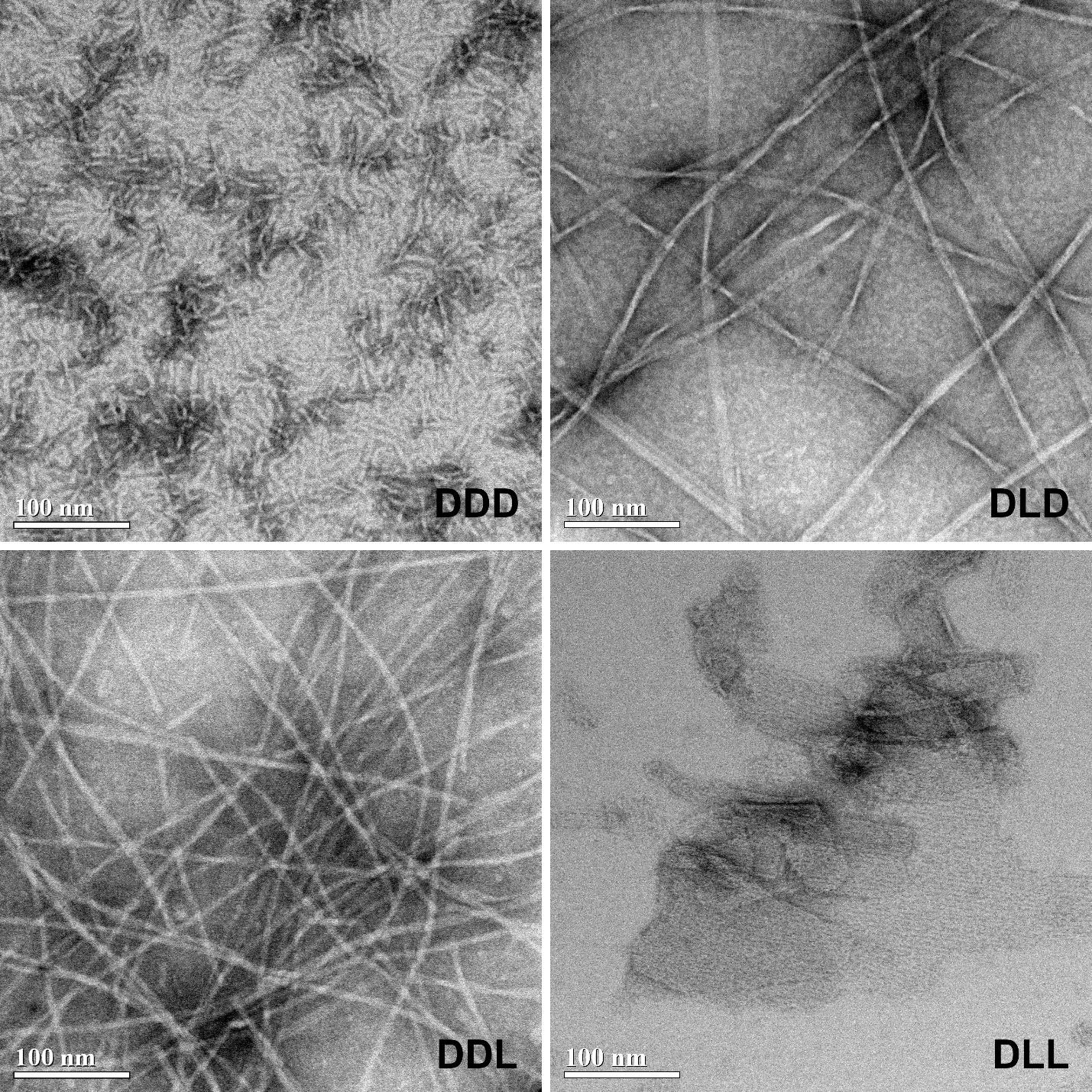


**Figure S1**. Representative STEM images for DDD, DLD, DDL, and DLL KFE12 at 0.10 mM in water.


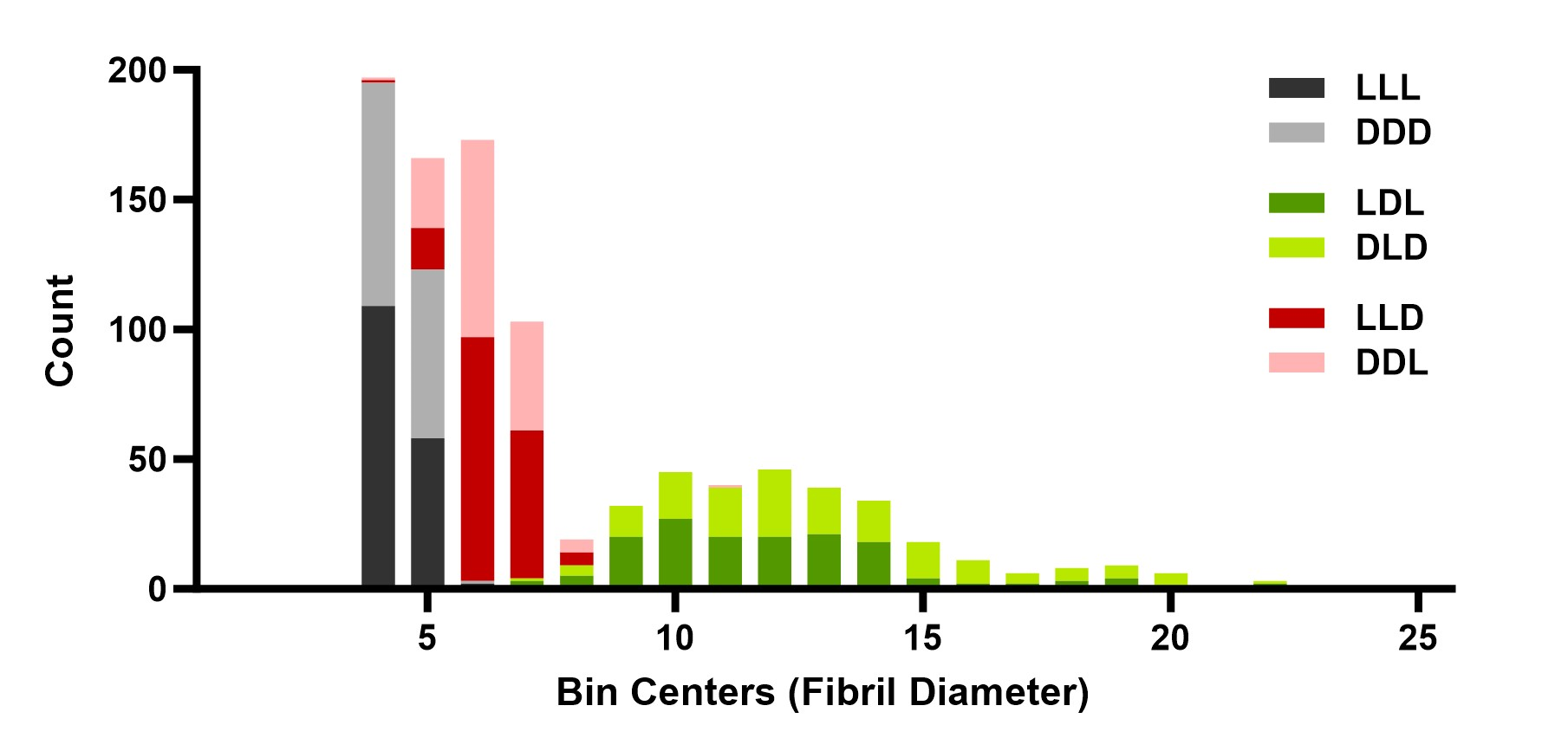


**Figure S2**. Histogram of KFE12 fibril width measurements. Data shown for LLL (4.4 ± 0.4 nm), DDD (4.5 ± 0.4 nm), LDL (11.9 ± 2.8 nm), DLD (13.0 ± 3.1 nm), LLD (6.3 ± 0.6 nm), and DDL (6.2 ± 0.8 nm). Minimum of n=150 for each analog.


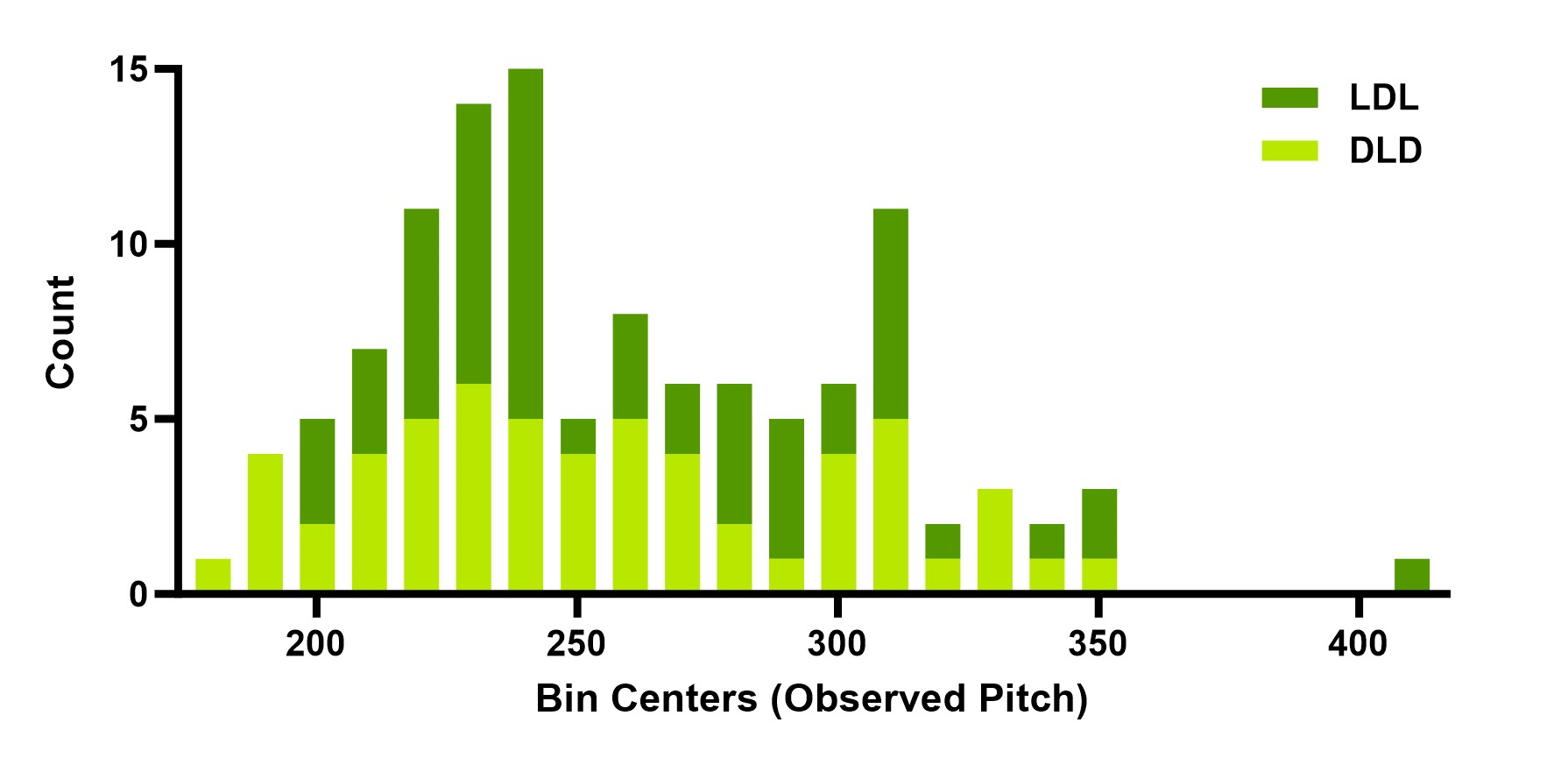


**Figure S3**. Histogram of LDL and DLD KFE12 fibril pitch measurements. Data shown for LDL (261 ± 45 nm) and DLD (257 ± 44 nm). Minimum of n=50 for each analog.


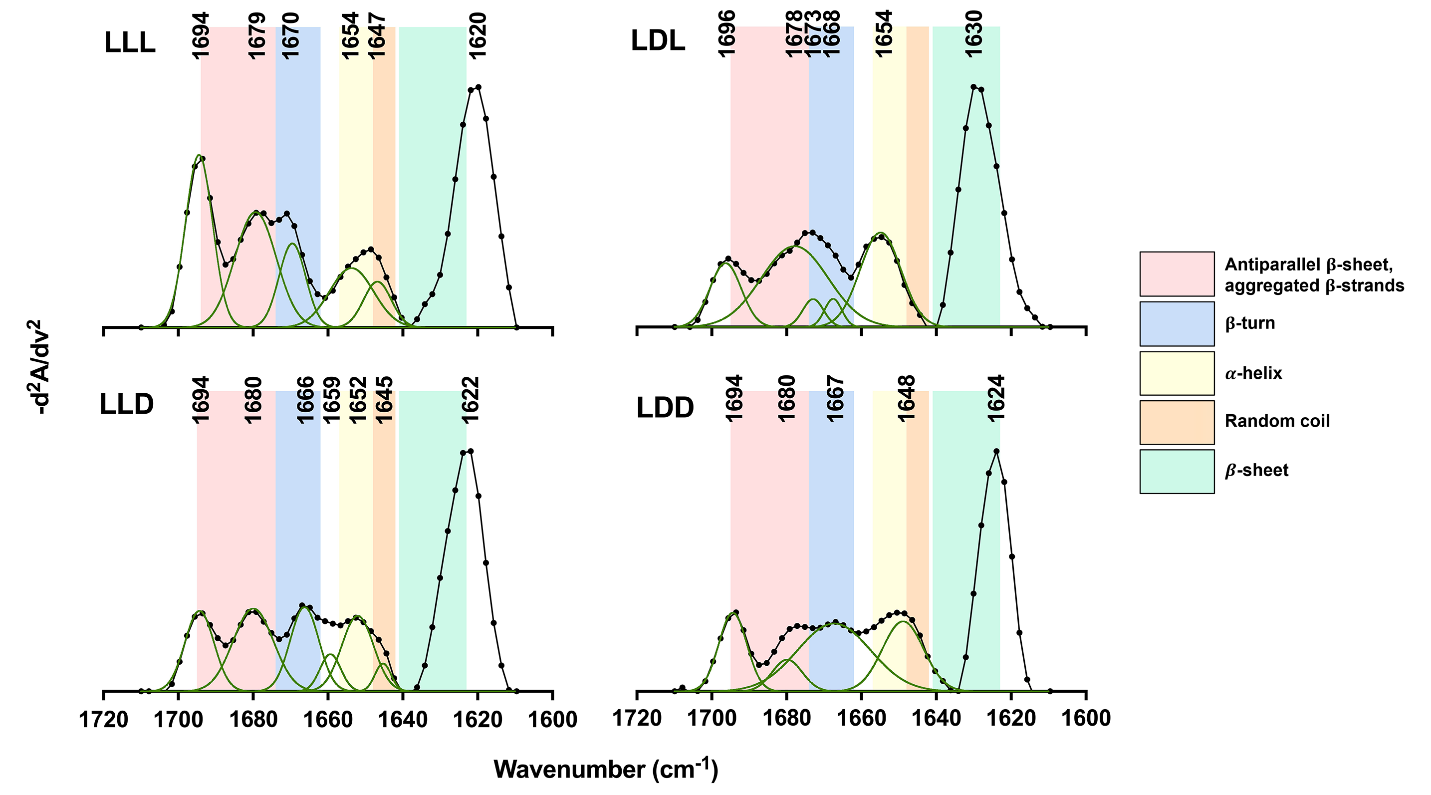


**Figure S4**. Second-derivative FTIR spectra and deconvolutions for LLL, LDL, LLD, and LDD. Experimental data shown in black, Gaussian components shown in green.


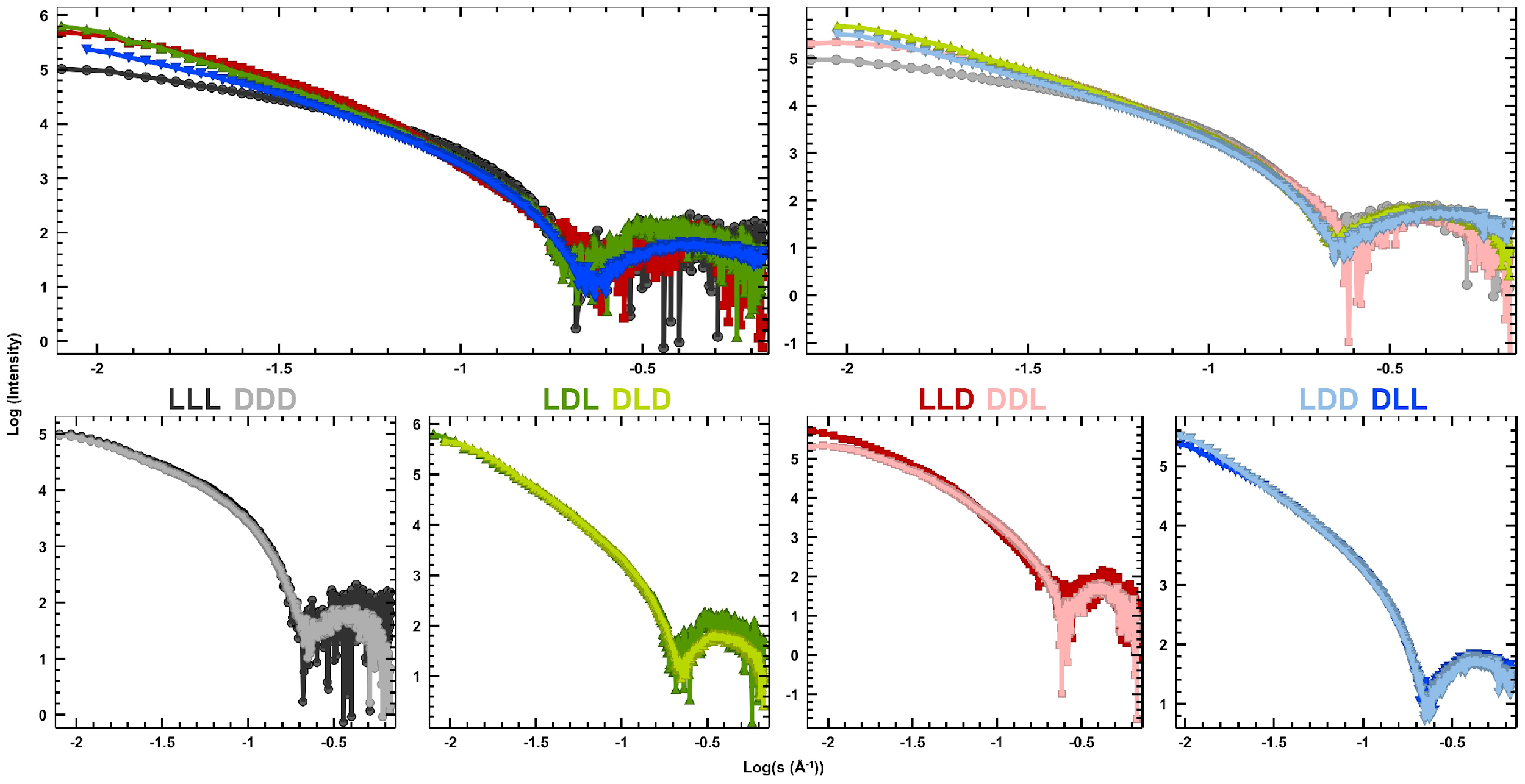
**Figure S5**. KFE12 SAXS data Log-Log plots. The L- and D-dominant analogs are shown in the top-left and top-right, respectively. Individual enantiomer pairs are also shown in the bottom row for clarity.


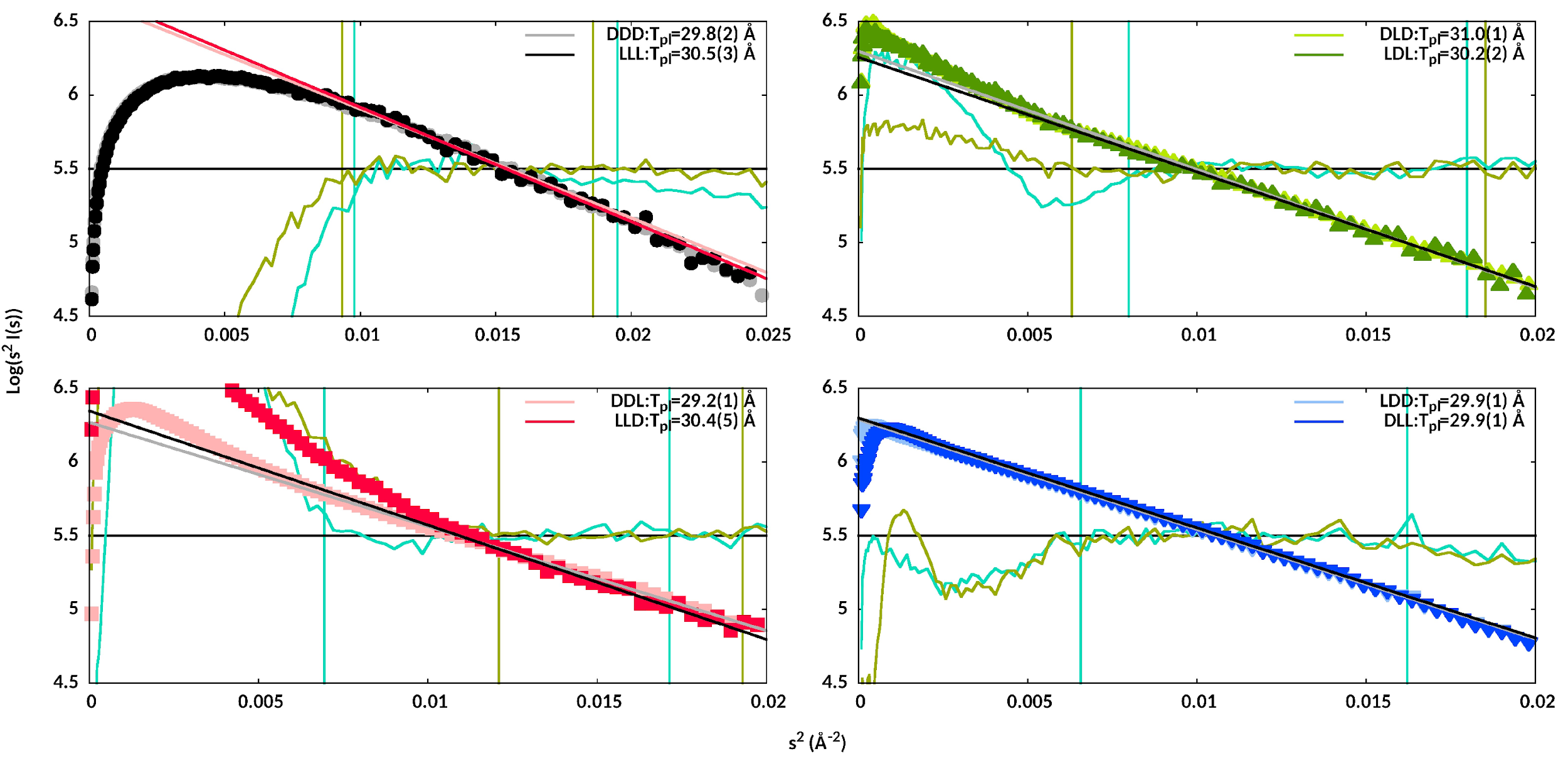
**Figure S6**. KFE12 Guinier fitting of the SAXS data to a thin plate of thickness T_p_. Enantiomer pairs are shown together with their Guinier fit. The residuals are shown in green for the L-dominant analogs and in teal for the D-dominant analogs. The fitting range (vertical lines) was determined by the best fit (~0.8/R_t_ to ~1.2/R_t_). Thickness values range from 29.2 to 31.0 Å.

**Table S1**. SAXS Characteristics of the Peptide Filaments and Gels

| **Peptide** | **Rg (Å)** | **Filament**  **R_cs_ (Å)** | **Thickness T_plate_ (Å)** | **Chain Length (Å)** | **Debye-Bueche (Å)** |
| --- | --- | --- | --- | --- | --- |
| **LLL** | 83* | 15* | 30.5(3) | 9000* | 15.6(1) |
| **DDD** | 83* | 15* | 29.8(2) | 300* | 15.6(1) |
| **LDL** | 141* | 48* | 30.2(2) | 4000* | 33(1) |
| **DLD** | 108* | 44* | 31.0(1) | 7000* | 30(2) |
| **LLD** | 121* | 43* | 30.4(5) | 1000* | 35.7(1) |
| **DDL** | 83* | 28* | 29.2(1) | 1000* | 28.4(3) |
| **LDD** | 128* | 33* | 29.9(1) | 1000* | 39(2) |
| **DLL** | 123* | 32* | 29.9(1) | 1000* | 32(1) |

*Curves not linear/value not well-defined

Table lists the Guinier radius of gyration (R_g_), filament Guinier cross-sectional radius (R_cs_), Guinier sheet thickness (R_t_, T_plate_=R_t_*√12), Gaussian chain correlation length, and Debye-Bueche heterogeneity parameter for each peptide.


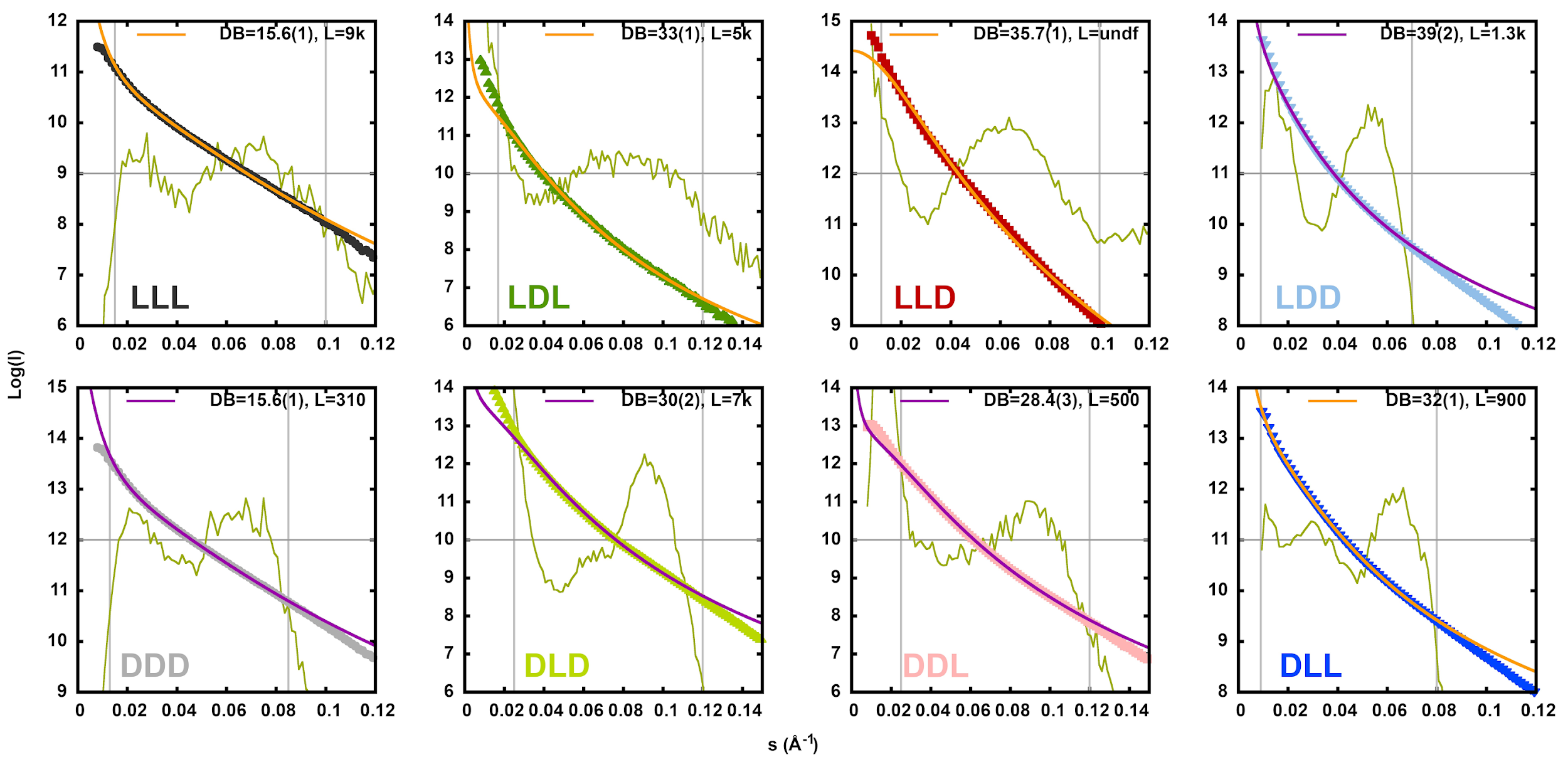
**Figure S7**. Debye-Bueche fitting of KFE12 SAXS data. The fitting range (vertical lines) was determined by best fit (±3σ). The Lorentzian contribution was ill-defined for L>1000 Å. Debye-Bueche heterogeneity factors range from 16 to 39 Å (Figure 4, Table S1).

**Table S2**. Summary of the WAXS Data Collection and Results

| **Peptide** | **LLL** | **DDD** | **LDL** | **DLD** | **LLD** | **DDL** | **LDD** | **DLL** |
| --- | --- | --- | --- | --- | --- | --- | --- | --- |
| **mg/mL** | 17 | 17 | 17 | 17 | 17 | 17 | 17 | 17 |
| **s_min_ (Å^-1^)** | 0.7^b^ | 0.7^b^ | 0.7^b^ | 0.7^b^ | 0.7^b^ | 0.7^b^ | 0.7^b^ | 0.7^b^ |
| **s_max_ (Å^-1^)** | 5.36/6.72 | 5.36/6.72 | 5.36/6.72 | 5.36/6.72 | 5.36/6.72 | 5.36/6.72 | 5.36/6.72 | 5.36/6.72 |
| **Peak (Å)** | 4.720 | 4.725 | 4.714 | 4.713 | 4.733 | 4.734 | 4.712 | 4.714 |
| **Peak (Å)** | - | - | - | - | 4.636 | 4.634 | 4.620 | 4.623 |
| **Peak (Å)** | 4.588 | 4.586 | 4.598 | 4.599 | - | - | - | - |
| **Peak (Å)** | - | - | 4.502 | 4.512 | 4.535 | 4.532 | 4.520 | 4.523 |
| **Peak (Å)** | - | - | - | - | - | - | 5.626 | 5.651 |
| **Peak (Å)** | - | - | 9.253 | 9.368 | 9.337 | 9.171 | 9.300 | 9.274 |
| **Peak (Å)** | - | - | 4.339 | 4.240 | - | - | 4.345 | 4.307 |

^b^The Beamstop shadow is diffuse and the start of unaffected data is uncertain. Peaks are sorted by relative intensity (high to low).


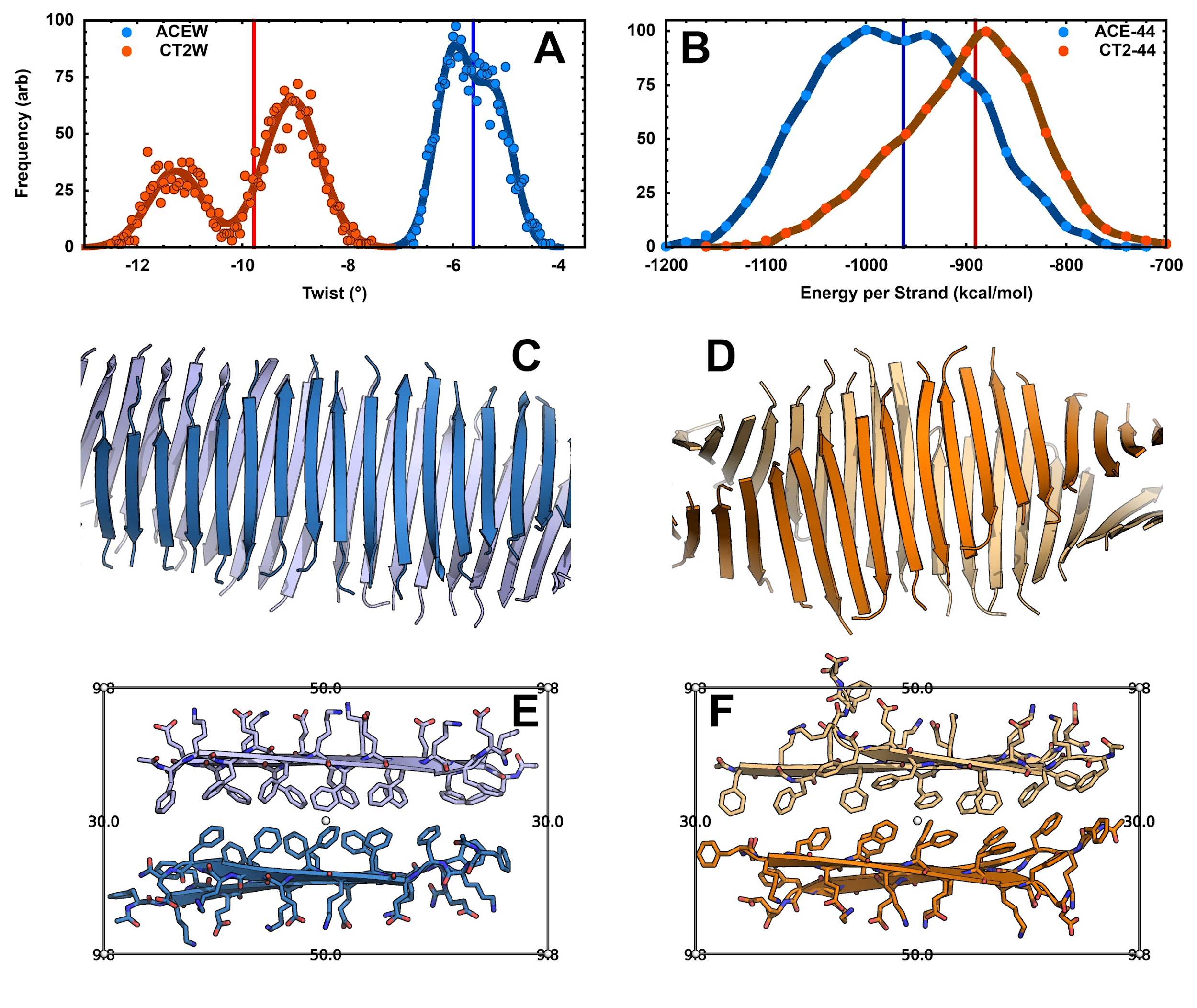


**Figure S8**. (A) Filament twist distribution of the LLL models (final 5 ns). Mean twist was calculated as -5.6° for the ACE model and -9.8° for the CT2 model. (B) NAMD free energy distributions of the two LLL models (ACE● and CT2●), ΔE = -77 kcal/mol/strand. (C) The ACE model after 10 ns of MD. (D) The CT2 LLL model after 10 ns of MD. (E,F) The filament a.u. for each model. The filament thickness of 30 Å (SAXS), is represented by the box height. The u.c. bounding box is 30 x 50 x 9.48 Å. The ACE filament is represented by a bounding box translated along the z-axis and rotated -11.2°.


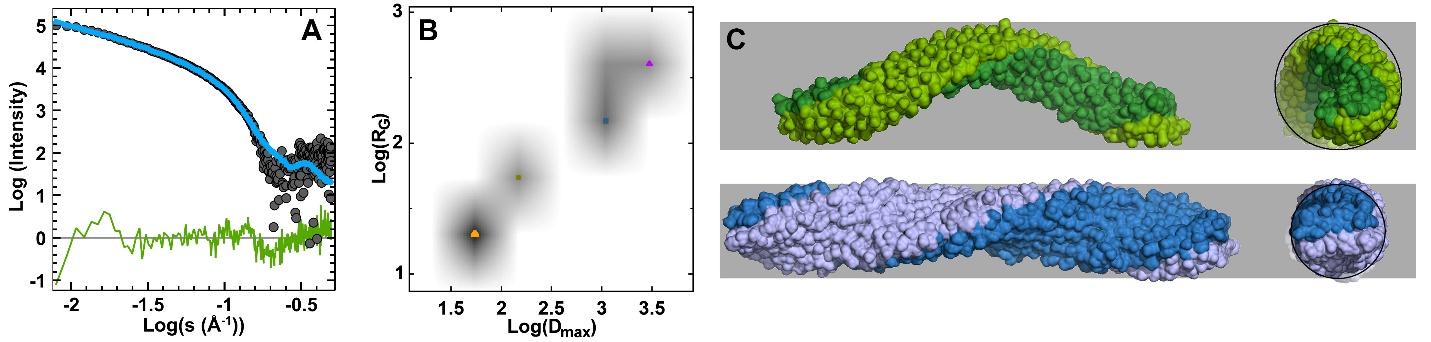


**Figure S9**. Energy-minimization modeling of LLL KFE12. (A) Fitting of the LLL SAXS data (χ^2^=2.1) with (B) an ensemble of model filaments, from small to moderately large (D_max_ up to 2500 Å). (C) Model filaments of LL KFE8 (green) and LLL KFE12 (blue). SAXS-derived fibril diameters are illustrated by the gray rectangles (background) and circles (right). The KFE8 model represents approximately a half-turn, while the KFE12 model is one full turn.


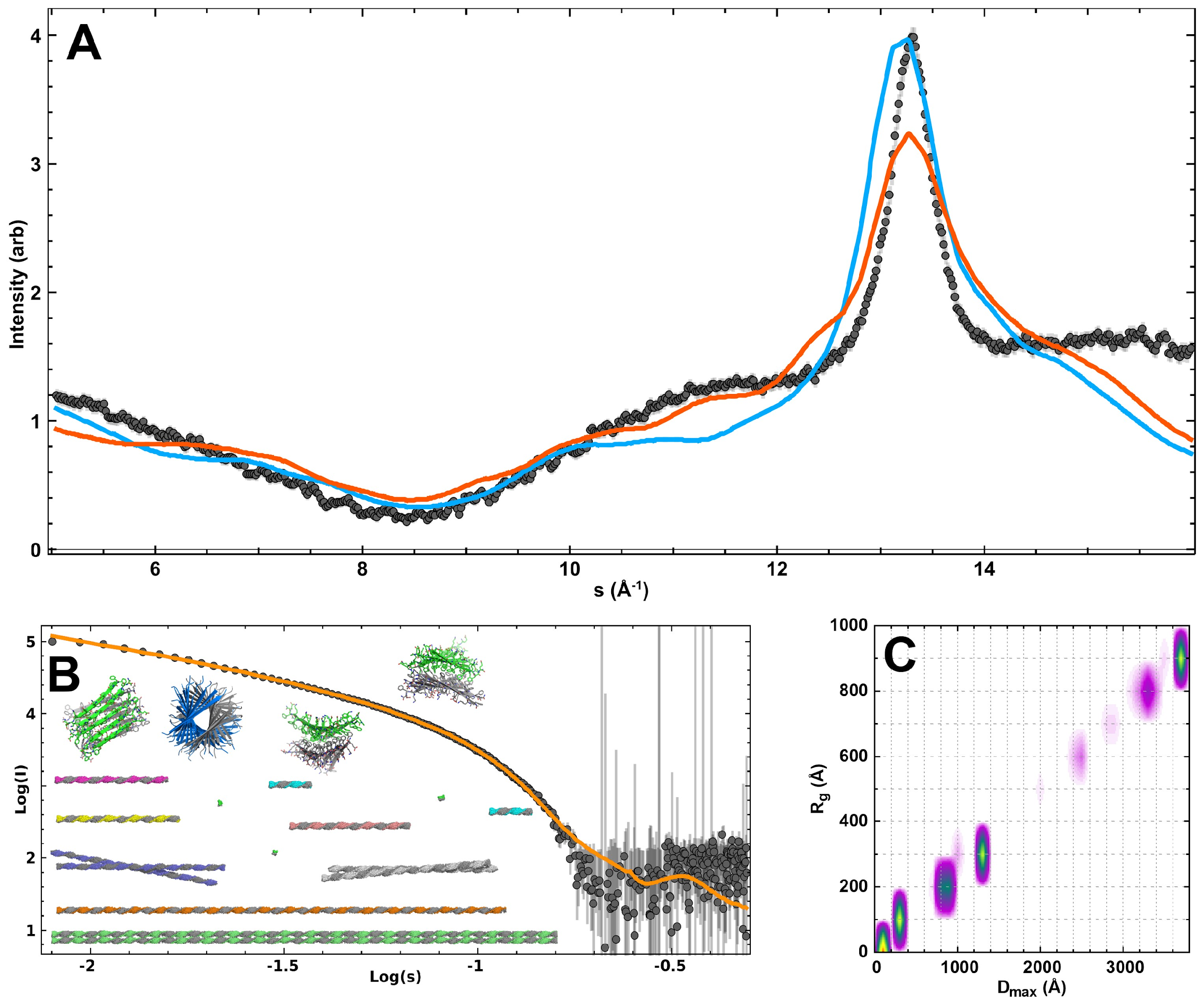
**Figure S10**. SAXS and WAXS ensemble fitting to the LLL filament MD models. (A) The WAXS data fit with two short (2x22 strand) MD filament models ACE(▬) and CT2(▬). The major features of the near-WAXS curve, the dip at s~8.4 (Å^-1^) and the β-sheet peak at s~13.3 (Å^-1^), are replicated in the scattering from both filament models. (B) The EOM ensemble fitting of filament models to LLL (χ^2^=1.7). The filament models selected by EOM are shown. (C) EOM distribution 2-D plot. The color scale indicates relative frequency of selection from none (white), to infrequent (purple), to more frequent (blue, green, yellow), to most frequent (red).


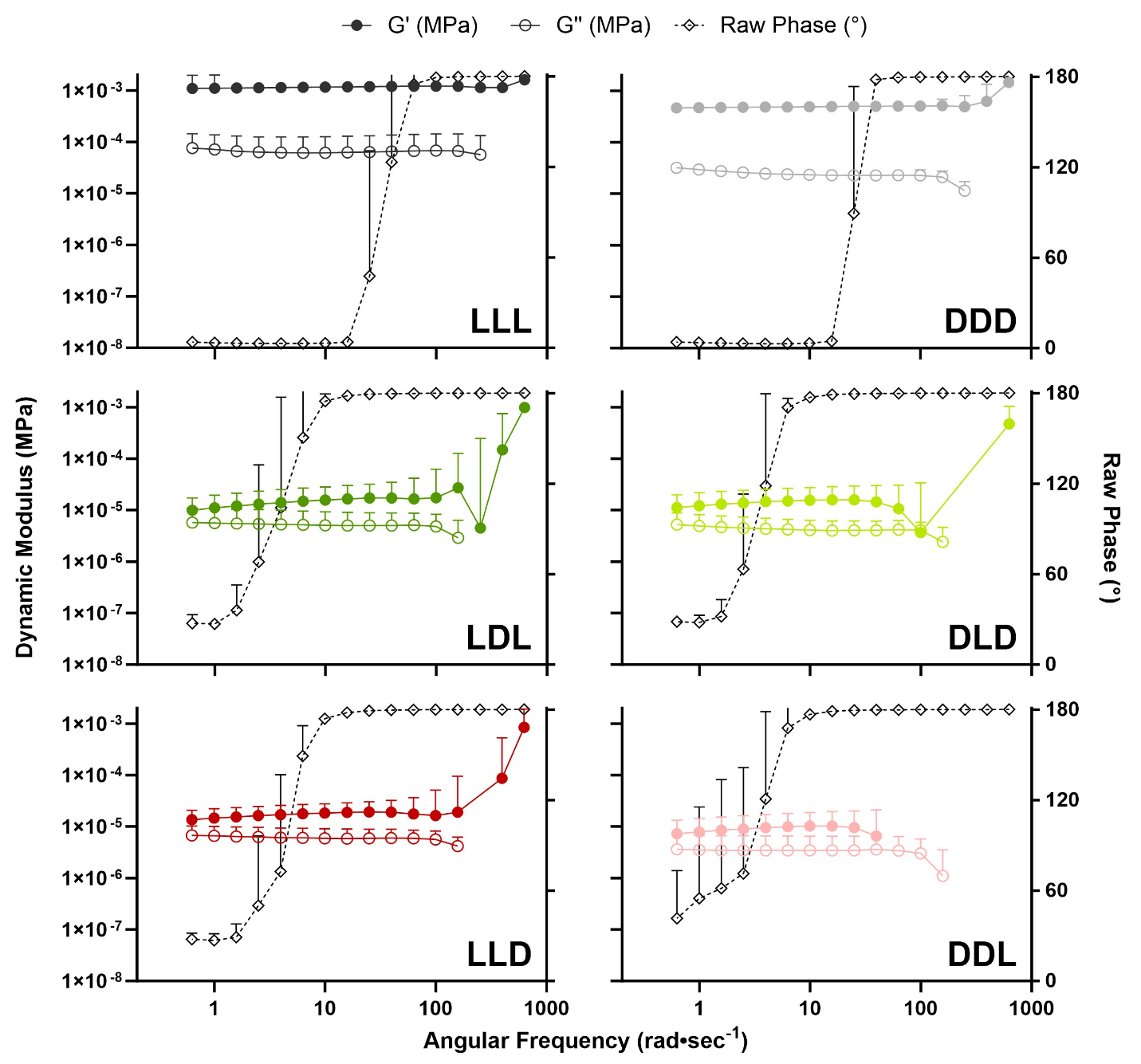


**Figure S11.** Frequency sweeps for LLL, DDD, LDL, DLD, LLD and DDL hydrogels (10 mM). Dynamic modulus is shown on left y-axis and raw phase on the right y-axis. For datapoints corresponding to raw phases lower than ~180°, the data is clearly valid, while datapoints corresponding to raw phases with high raw phases may include measurement artifacts. Error bars represent the mean±SEM of n=3 gels per sample.


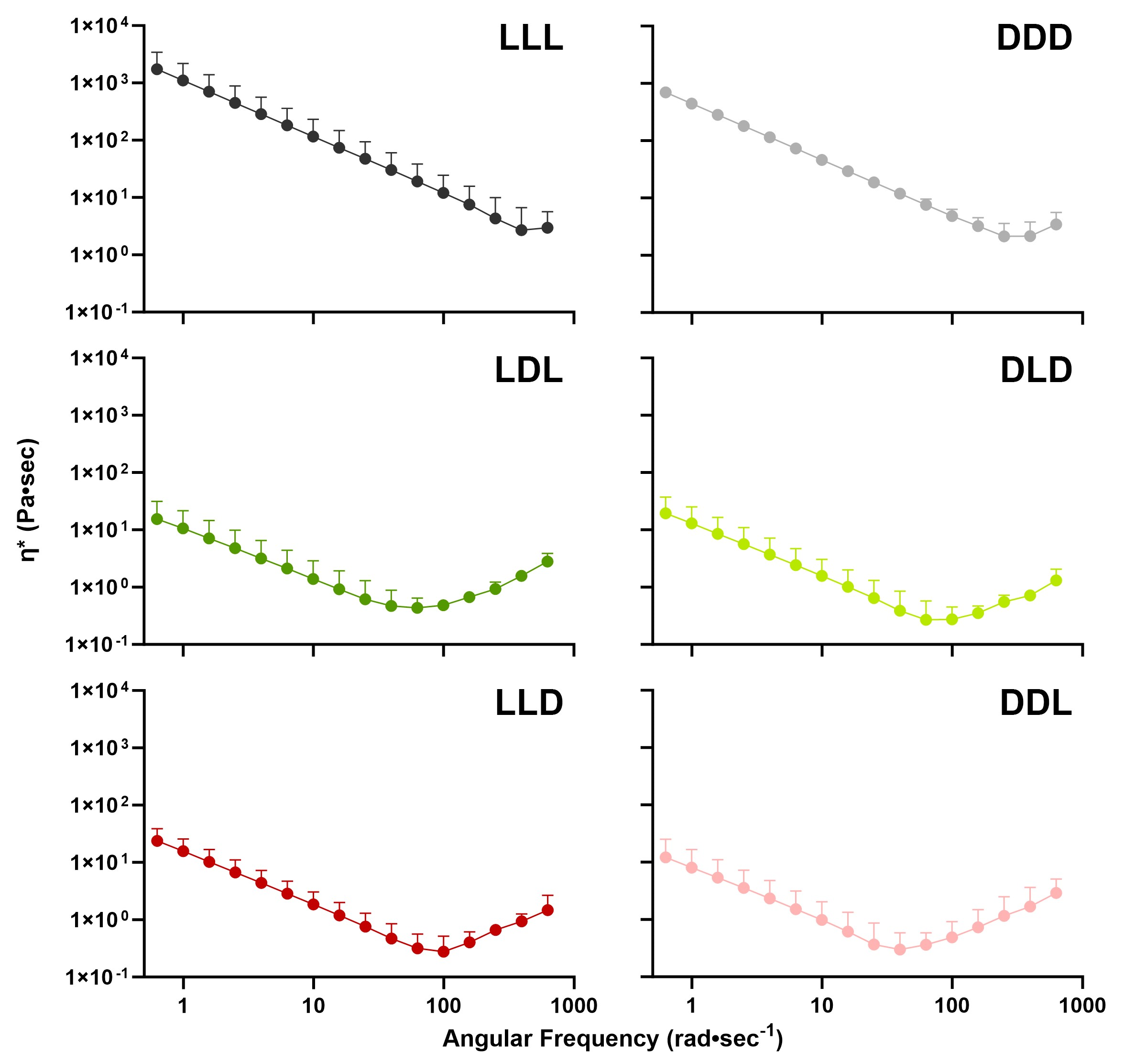


**Figure S12.** Complex viscosity as calculated based on frequencies and dynamic moduli from frequency sweeps for LLL, DDD, LDL, DLD, LLD and DDL hydrogels (10 mM). The downward trend indicates that the gels are shear thinning. Error bars represent the mean±SEM of n=3 gels per sample.


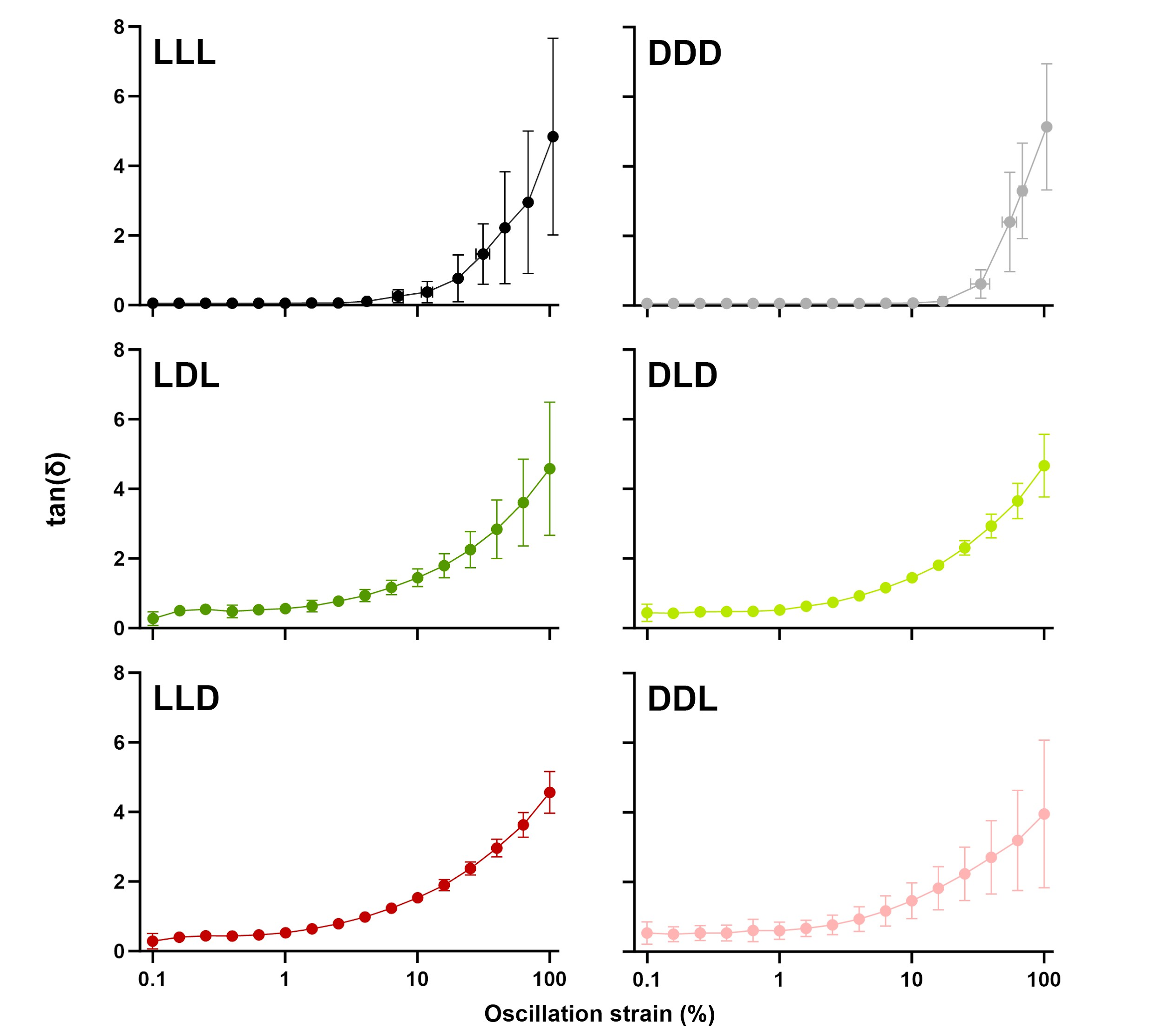


**Figure S13.** Phase angle determined during amplitude sweeps for LLL, DDD, LDL, DLD, LLD and DDL hydrogels (10 mM). In the range where tan(δ) remains approximately constant, the gel is within its linear range. The homochiral peptides tend to have linear ranges that extend to substantially higher oscillatory strains than the heterochiral peptides. Error bars represent the mean±SEM of n=3 gels per sample.

**Table S3**. Peptide mass and purity.

| **Peptide** | **Calculated M (Da)** | **Observed MH^+^ (Da)** | **Purity (%)** |
| --- | --- | --- | --- |
| **LLL** | 1713.97 | 1735.86* | 94.88 |
| **DDD** | 1713.97 | 1713.86 | 97.69 |
| **LDL** | 1713.97 | 1713.91 | 96.71 |
| **DLD** | 1713.97 | 1713.89 | 96.43 |
| **LLD** | 1713.97 | 1713.83 | 95.07 |
| **DDL** | 1713.97 | 1713.88 | 97.96 |
| **LDD** | 1713.97 | 1713.84 | 97.00 |
| **DLL** | 1713.97 | 1713.87 | 95.31 |

*MNa^+^ observed for LLL

Table shows the calculated and observed masses, and purities for the characterized peptides.

**
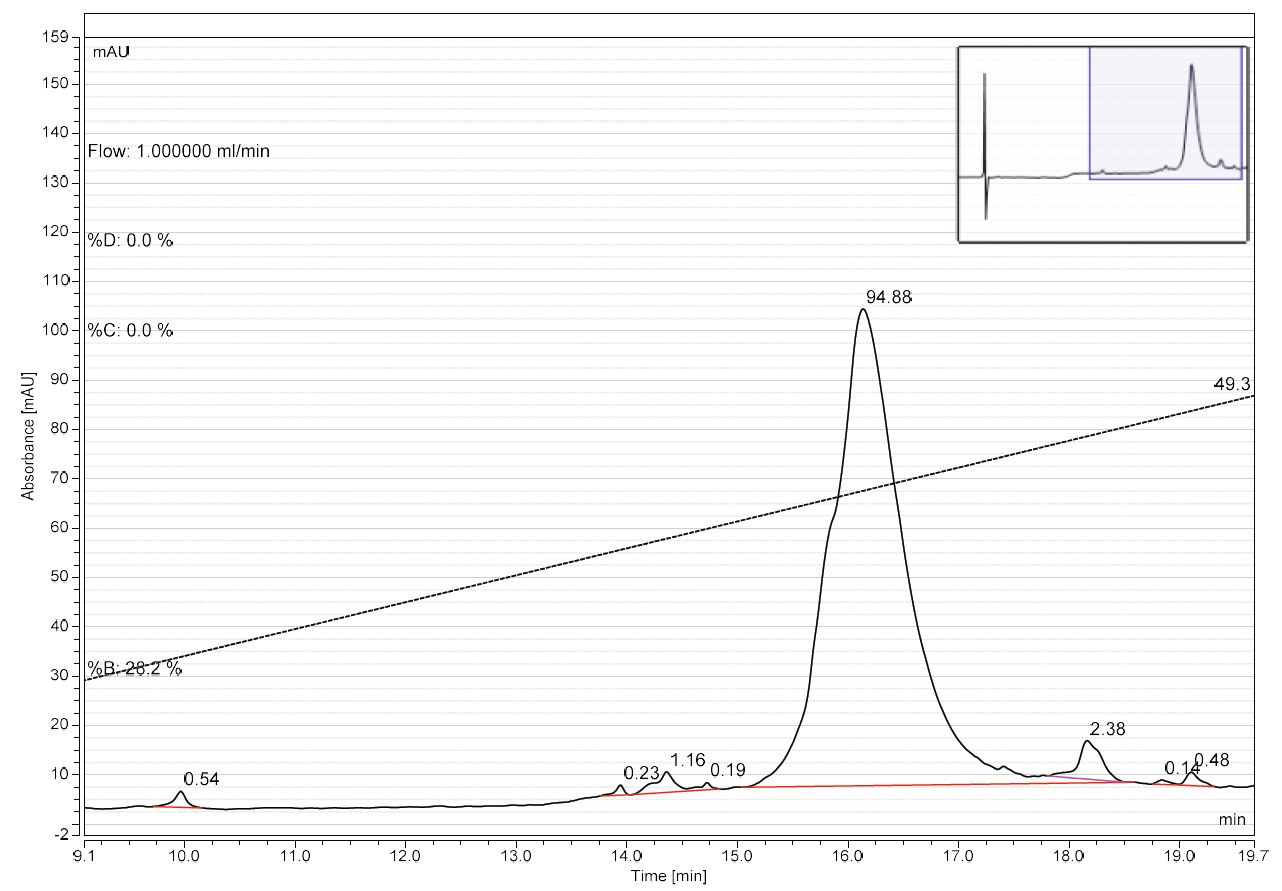
**

**Figure S14.** LLL peptide HPLC chromatogram.


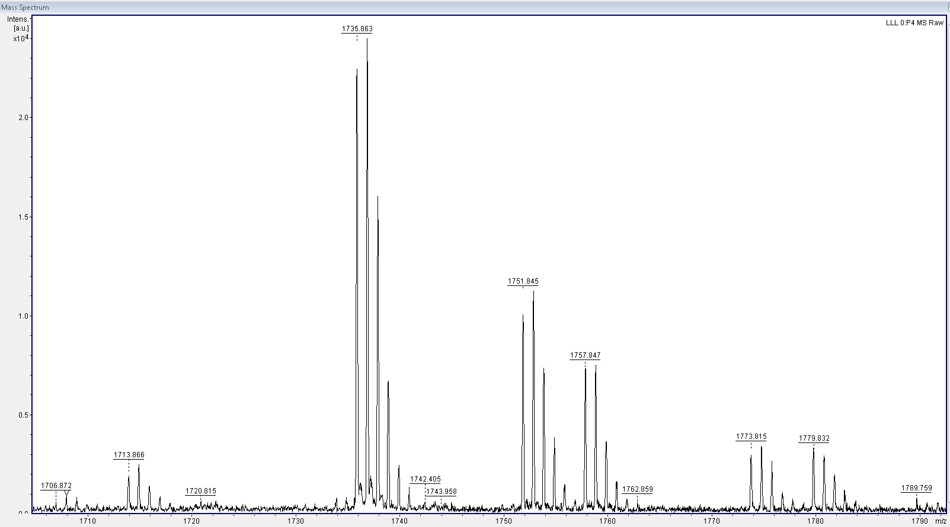


**Figure S15**. LLL peptide MALDI-TOF-MS spectrum.

**
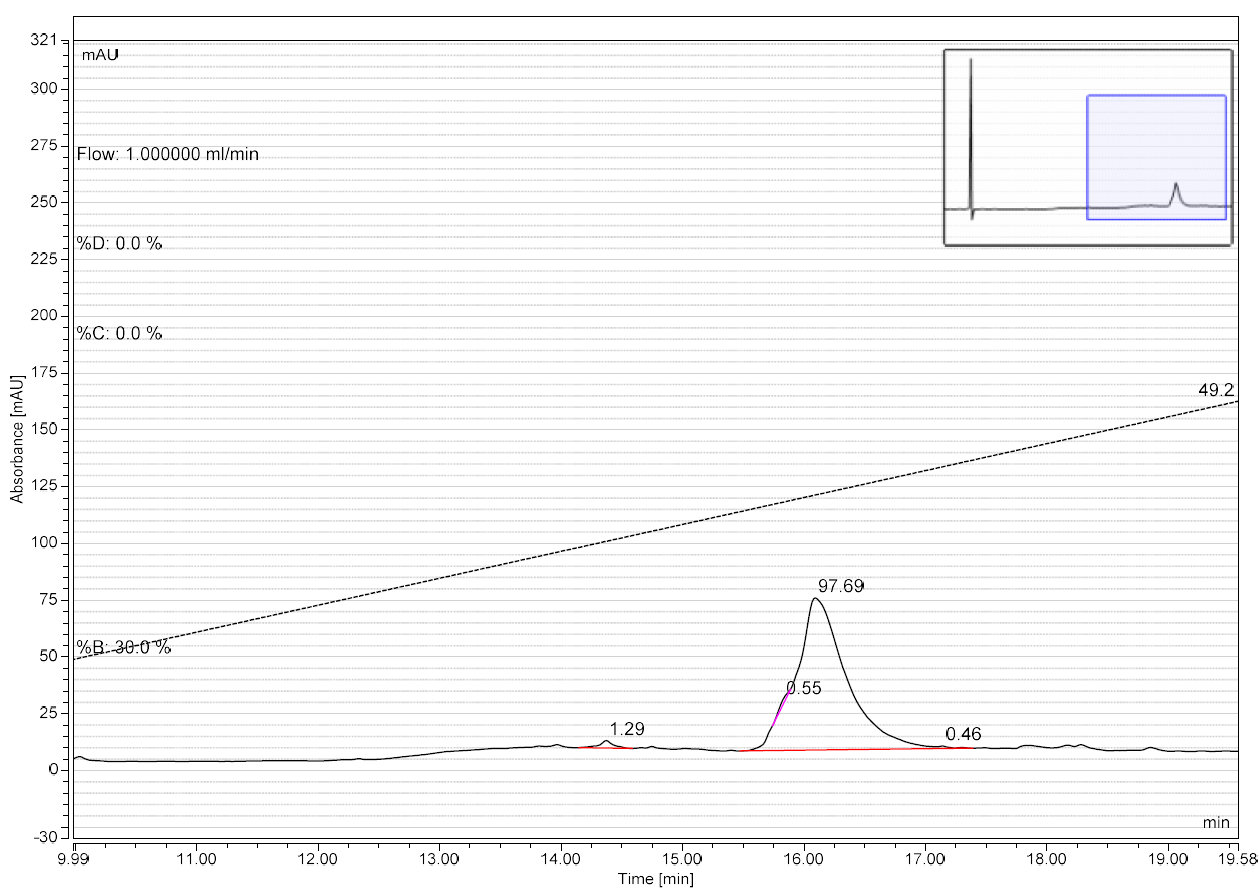
**

**Figure S16.** DDD peptide HPLC chromatogram.


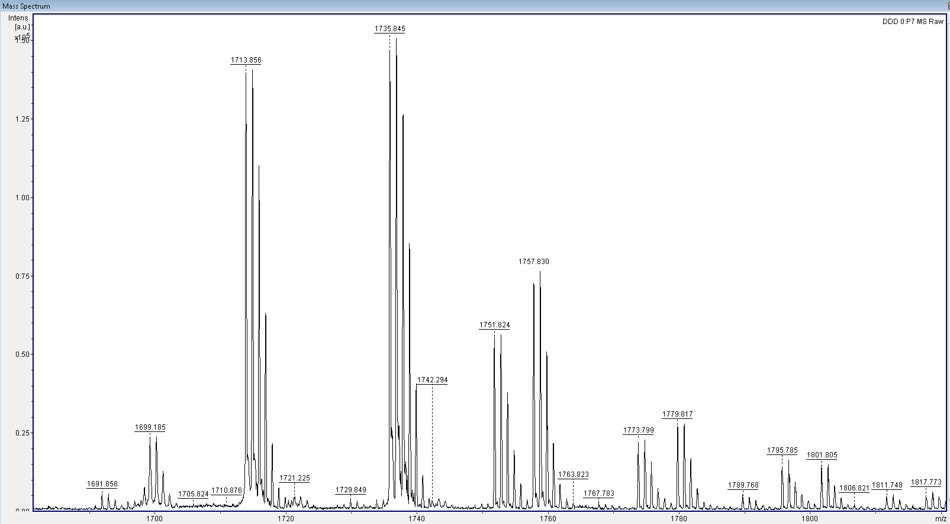


**Figure S17**. DDD peptide MALDI-TOF-MS spectrum.

**
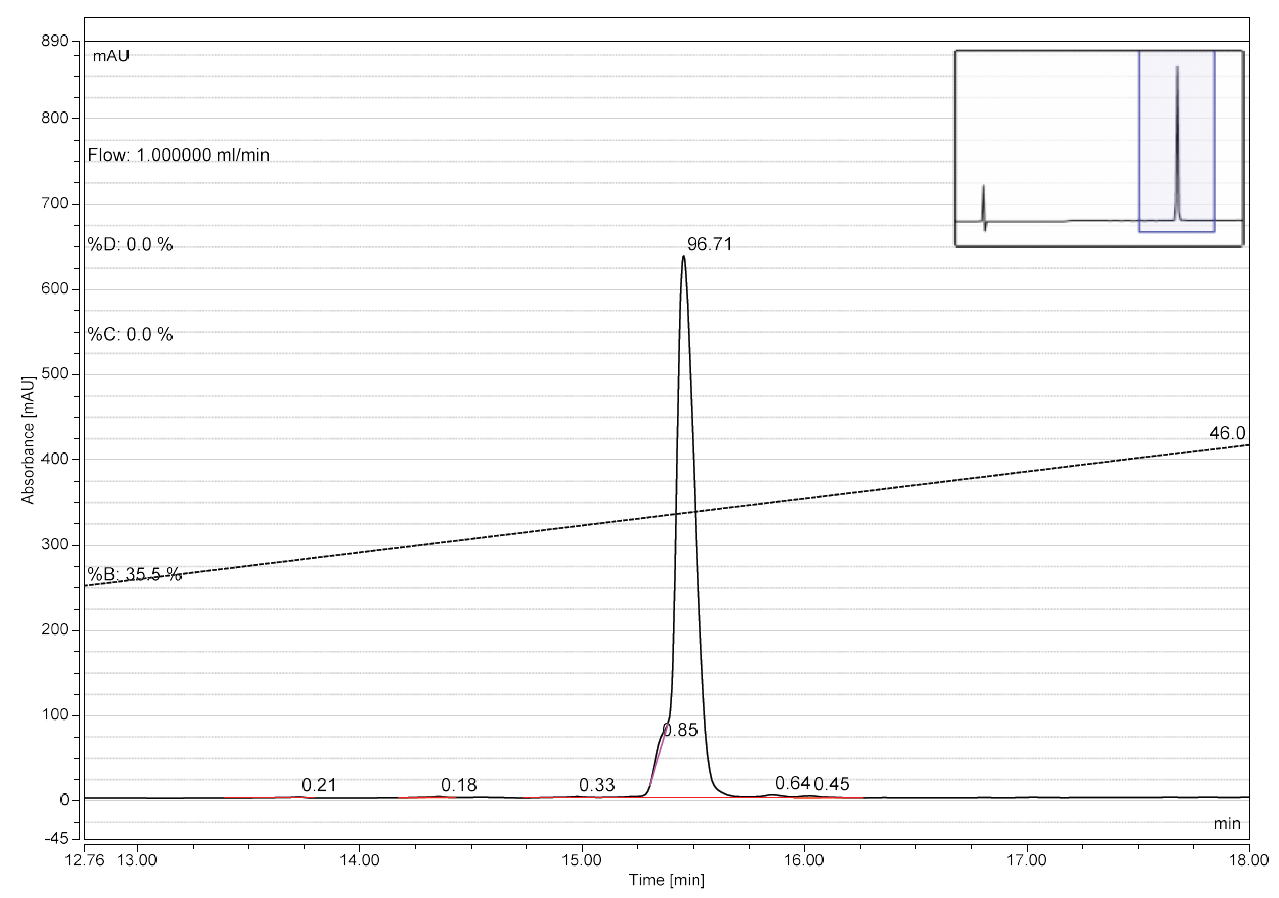
**

**Figure S18.** LDL peptide HPLC chromatogram.


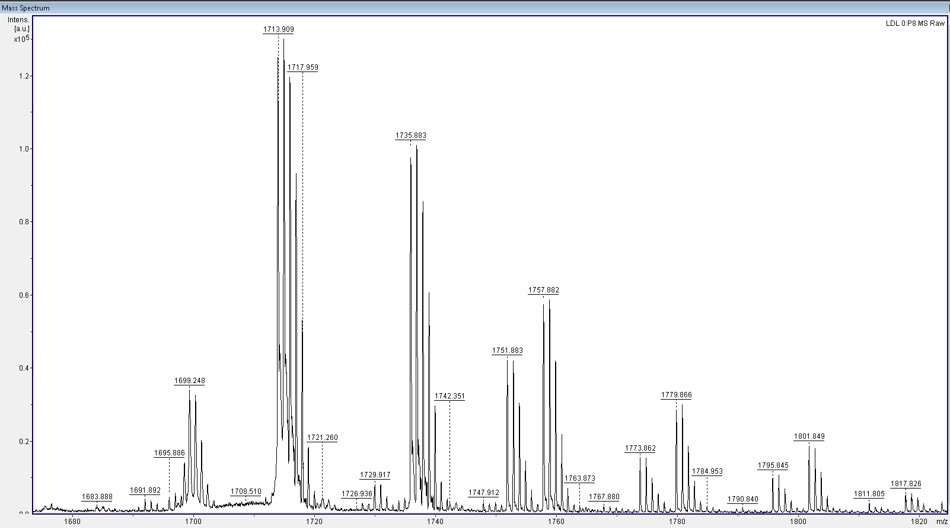


**Figure S19**. LDL peptide MALDI-TOF-MS spectrum.

**
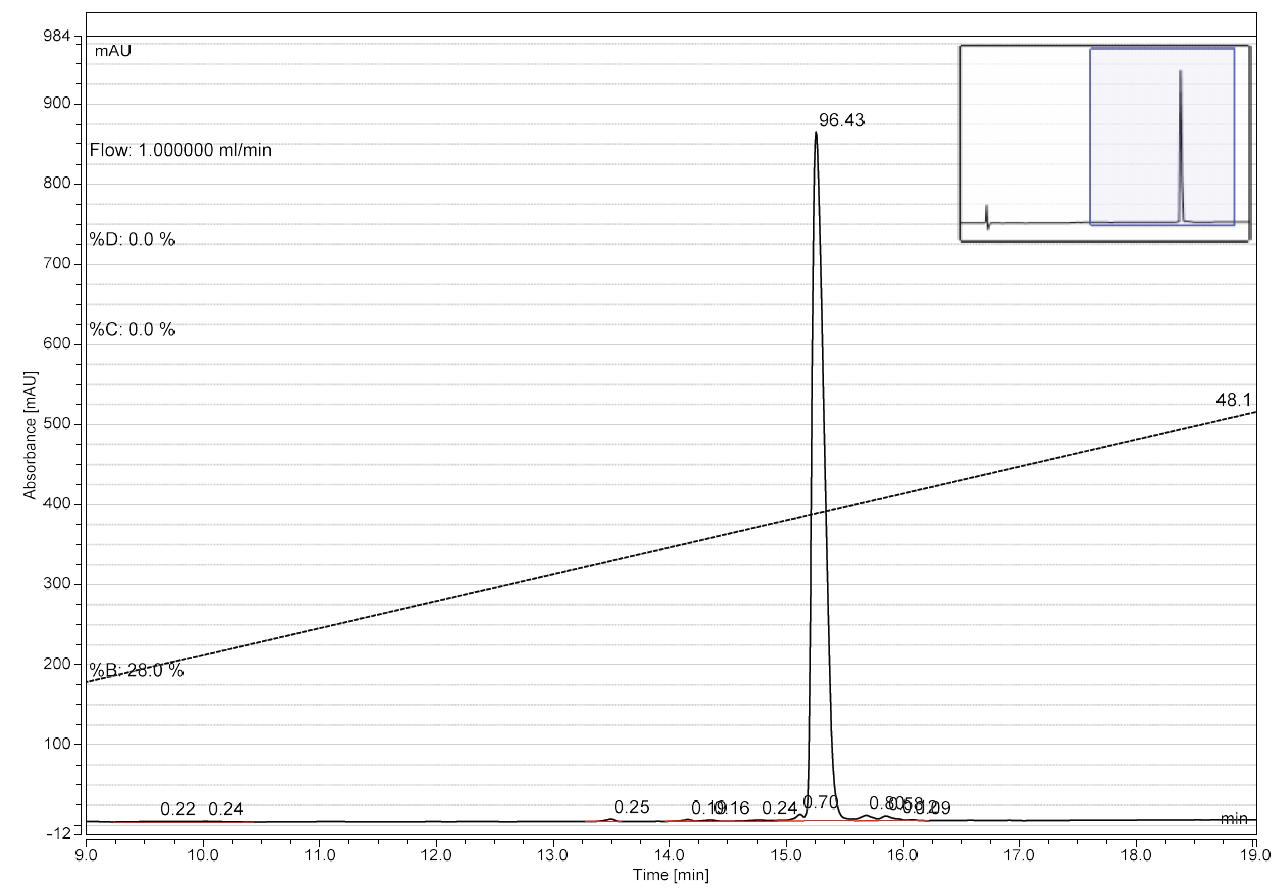
**

**Figure S20.** DLD peptide HPLC chromatogram.


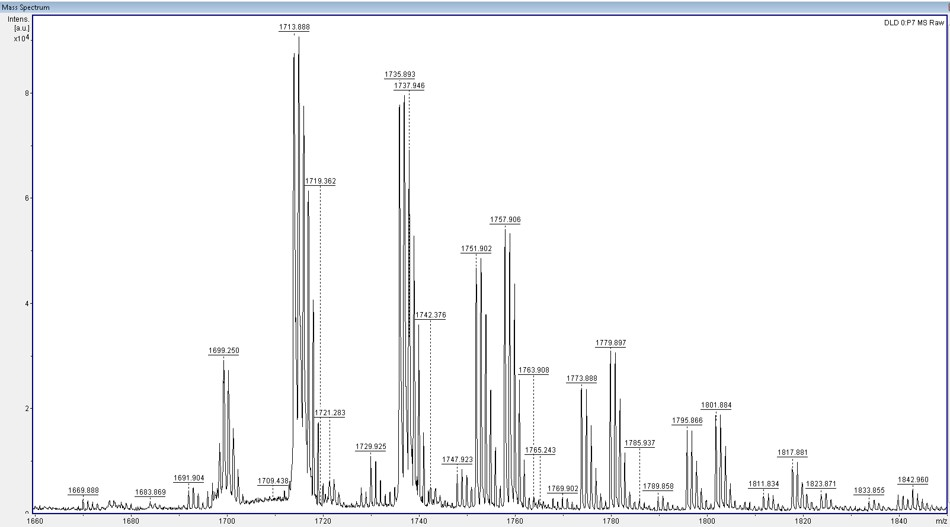


**Figure S21**. DLD peptide MALDI-TOF-MS spectrum.


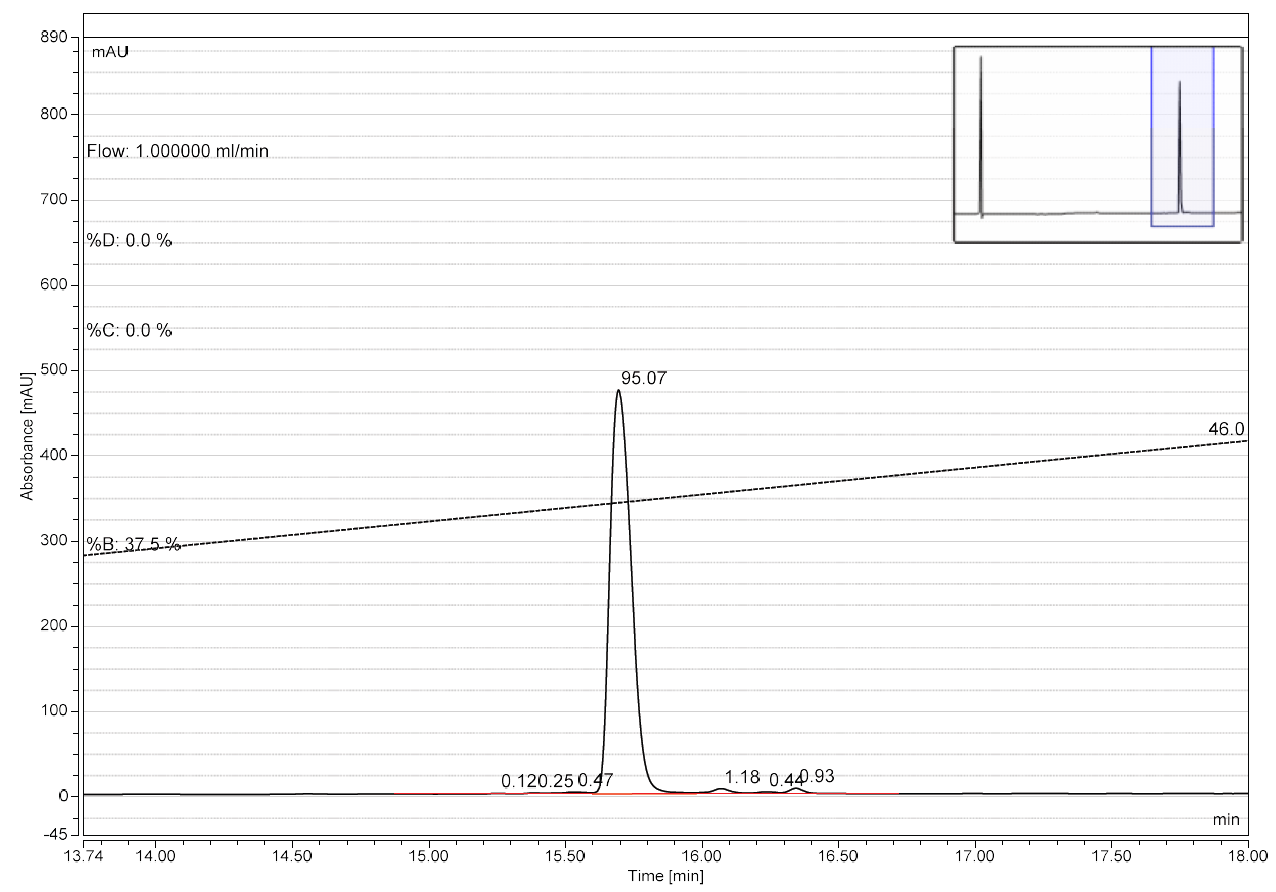


**Figure S22.** LLD peptide HPLC chromatogram.


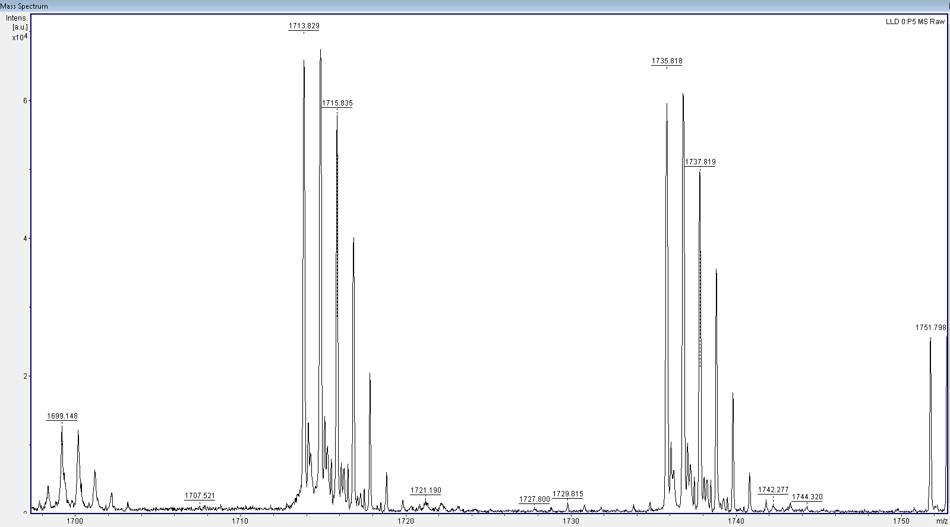


**Figure S23**. LLD peptide MALDI-TOF-MS spectrum.


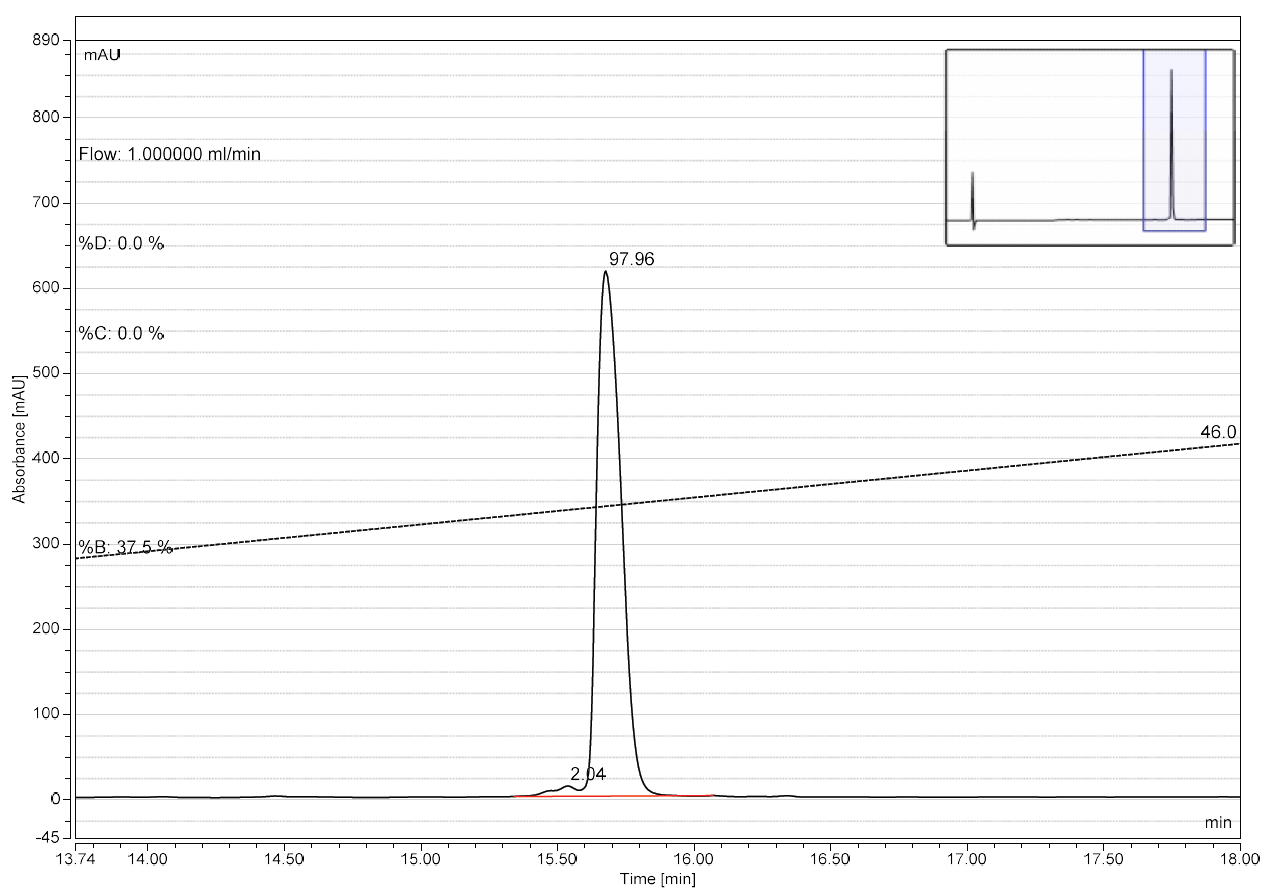


**Figure S24.** DDL peptide HPLC chromatogram.


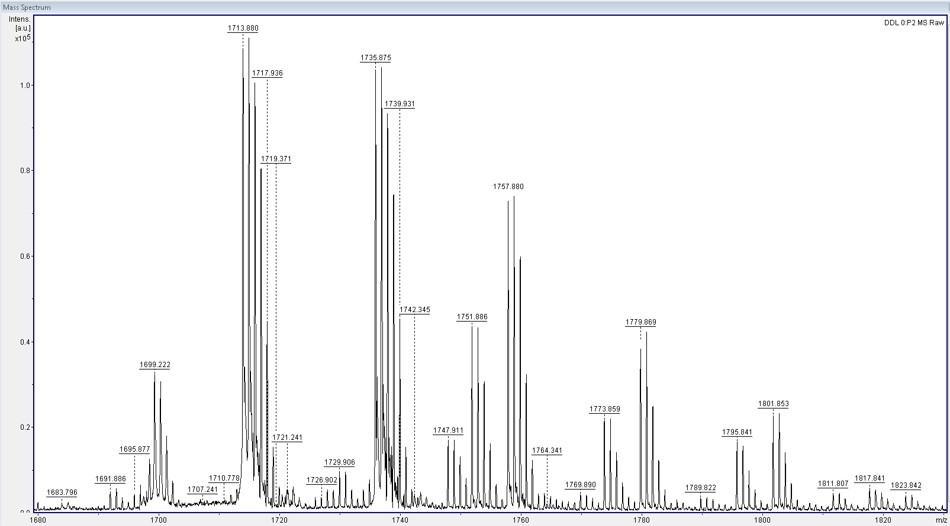


**Figure S25**. DDL peptide MALDI-TOF-MS spectrum.


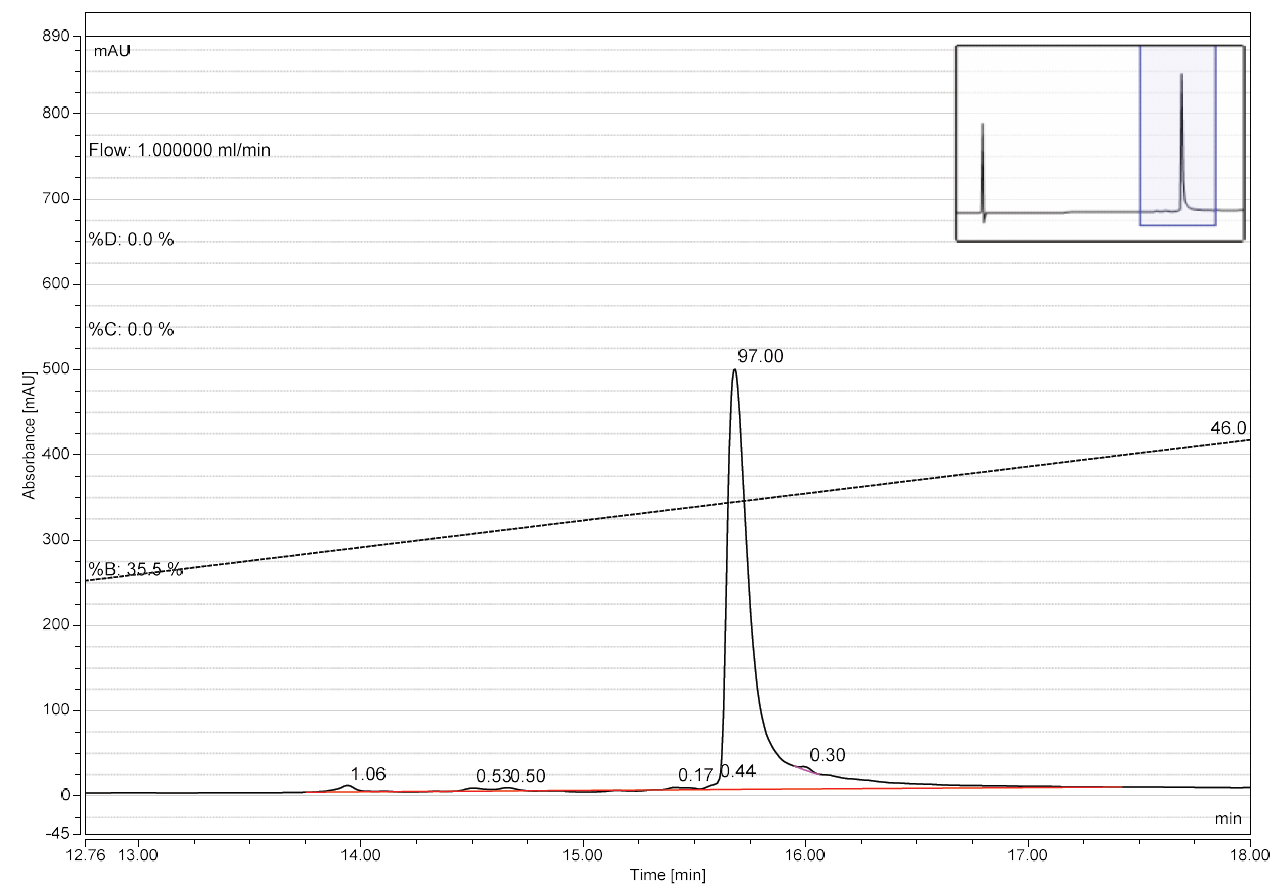


**Figure S26.** LDD peptide HPLC chromatogram.


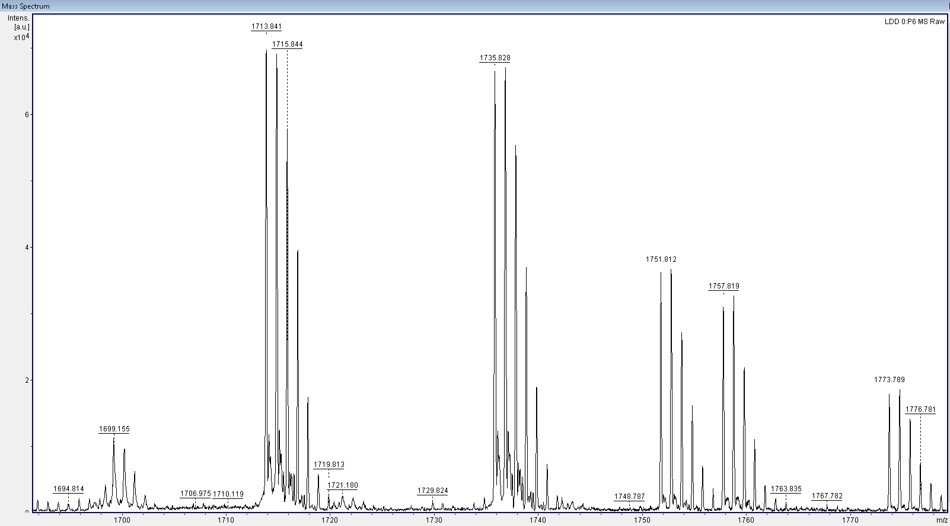


**Figure S27**. LDD peptide MALDI-TOF-MS spectrum.

**
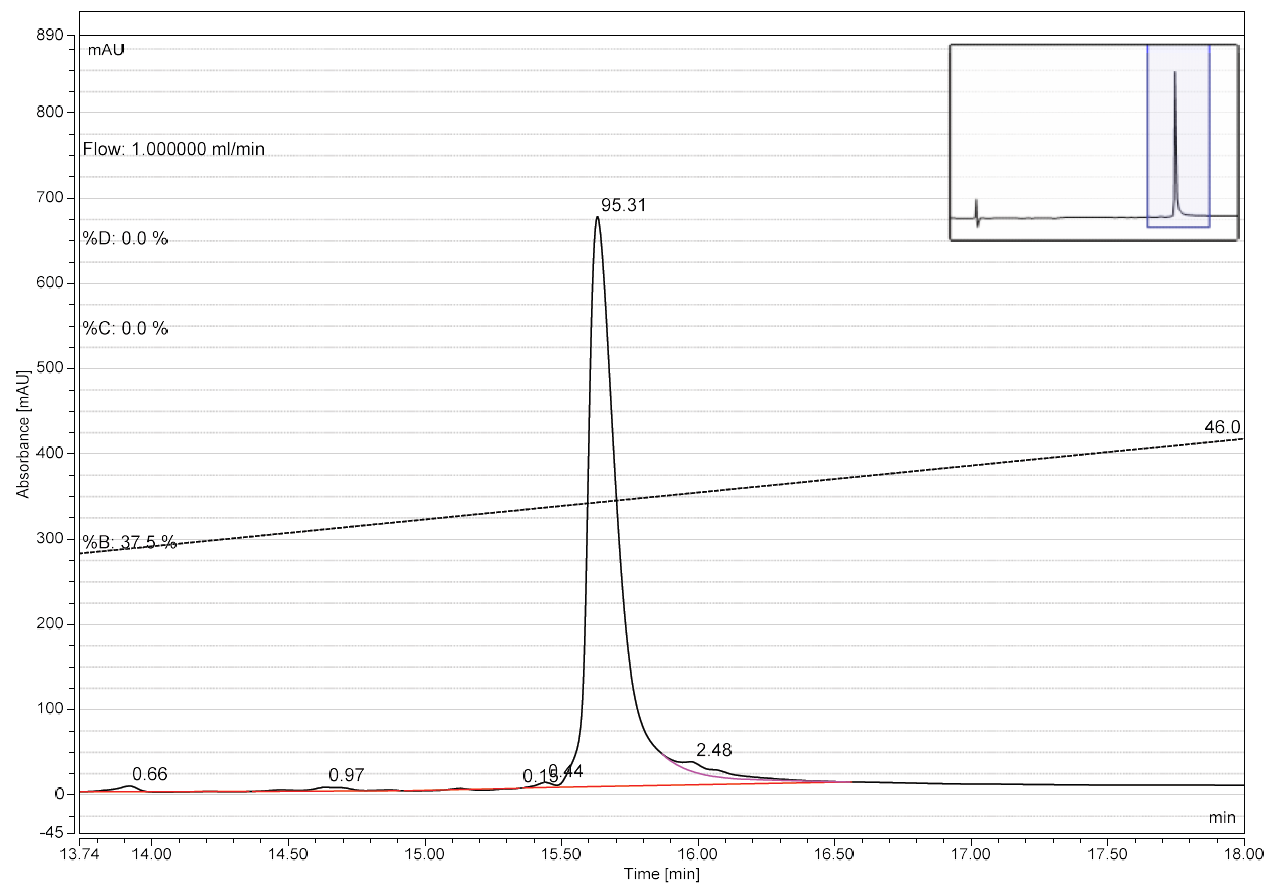
**

**Figure S28.** DLL peptide HPLC chromatogram.


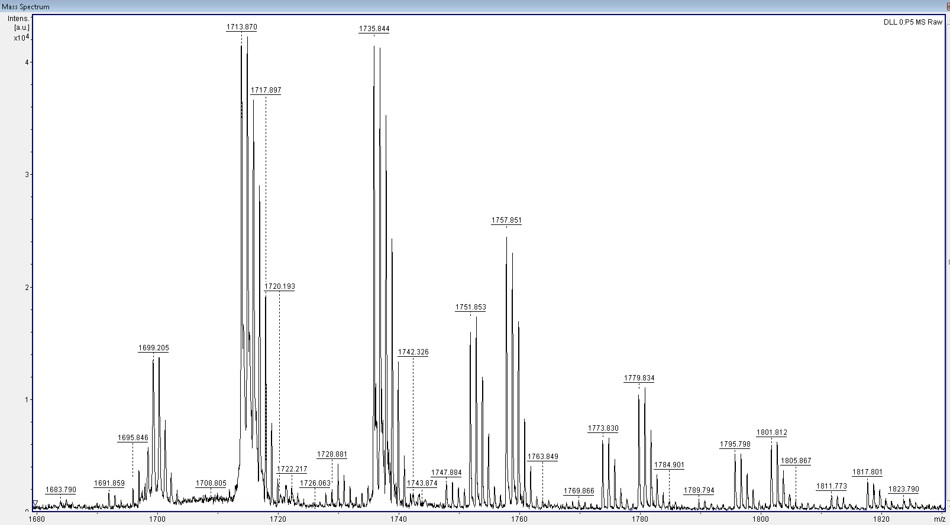


**Figure S29**. DLL peptide MALDI-TOF-MS spectrum.

REFERENCES

[1] A. Barth, *Biochim. Biophys. Acta BBA - Bioenerg.* **2007**, *1767* (9), 1073–1101. https://doi.org/10.1016/j.bbabio.2007.06.004.

[2] A. Dong, P. Huang, W. S. Caughey, *Biochemistry* **1990**, *29* (13), 3303–3308. https://doi.org/10.1021/bi00465a022.

[3] M. Jackson, H. H. Mantsch, *Crit. Rev. Biochem. Mol. Biol.* **1995**, *30* (2), 95–120. https://doi.org/10.3109/10409239509085140.

[4] K. Manalastas-Cantos, P. V. Konarev, N. R. Hajizadeh, A. G. Kikhney, M. V. Petoukhov, D. S. Molodenskiy, A. Panjkovich, H. D. T. Mertens, A. Gruzinov, C. Borges, C. M. Jeffries, D. I. Svergun, D. Franke, *J. Appl. Crystallogr.* **2021**, *54* (1), 343–355. https://doi.org/10.1107/S1600576720013412.

[5] S. Hansen, *J. Appl. Crystallogr.* **2000**, *33* (6), 1415–1421. https://doi.org/10.1107/S0021889800012930.

[6] A. H. Larsen, M. C. Pedersen, *J. Appl. Crystallogr.* **2021**, *54* (5), 1281–1289. https://doi.org/10.1107/S1600576721006877.

[7] D. Svergun, C. Barberato, M. H. J. Koch, *J. Appl. Crystallogr.* **1995**, *28* (6), 768–773. https://doi.org/10.1107/S0021889895007047.

[8] G. Tria, H. D. T. Mertens,M. Kachala, D. I. Svergun, *IUCrJ* **2015**, *2* (2), 207–217. https://doi.org/10.1107/S205225251500202X.

[9] J.-H An, A. N. Kiyonga, W. Yoon, M. Park, C. Lim, Y. Yun, G.-H. Park, K. Jung, *Crystals* **2017**, *7* (10), 284. https://doi.org/10.3390/cryst7100284.

[10] G. M. Brown, H. A. Levy, *Acta Crystallogr. B* **1973**, *29* (4), 790–797. https://doi.org/10.1107/S0567740873003353.

[11] G. M. Brown, H. A. Levy, *Science* **1963**, *141* (3584), 921–923. https://doi.org/10.1126/science.141.3584.921.

[12] C. A. Beevers, T. R. R. McDonald, J. H. Robertson, F. Stern, *Acta Crystallogr.* **1952**, *5* (5), 689–690. https://doi.org/10.1107/S0365110X52001908.

[13] D. Schneidman-Duhovny, M. Hammel, A. Sali, *Nucleic Acids Res.* **2010**, *38* (Web Server issue), W540-544. https://doi.org/10.1093/nar/gkq461.

[14] C. J. Knight, J. S. Hub, *Nucleic Acids Res.* **2015**, *43* (W1), W225–W230.

[15] W. Hwang, D. M. Marini, R. D. Kamm, S. Zhang, *J. Chem. Phys.* **2003**, *118* (1), 389–397. https://doi.org/10.1063/1.1524618.

[16] J. C. Phillips, D. J. Hardy, J. D. C. Maia, J. E. Stone, J. V. Ribeiro, R. C. Bernardi, R. Buch, G. Fiorin, J. Hénin, W. Jiang, R. McGreevy, M. C. R. Melo, B. K. Radak, R. D. Skeel, A. Singharoy, Y. Wang, B. Roux, A. Aksimentiev, Z. Luthey-Schulten, L. V. Kalé, K. Schulten, C. Chipot, E. Tajkhorshid, *J. Chem. Phys.* **2020**, *153* (4), 044130. https://doi.org/10.1063/5.0014475.

[17] W. Humphrey, A. Dalke, K. Schulten, *J. Mol. Graph.* **1996**, *14* (1), 33–38, 27–28. https://doi.org/10.1016/0263-7855(96)00018-5.
